# Speciation rates are unrelated to the formation of population structure in Malagasy gemsnakes

**DOI:** 10.1002/ece3.10344

**Published:** 2023-07-28

**Authors:** Frank T. Burbrink, Sara Ruane, Nirhy Rabibisoa, Achille P. Raselimanana, Christopher J. Raxworthy, Arianna Kuhn

**Affiliations:** ^1^ Department of Herpetology American Museum of Natural History New York City New York USA; ^2^ Life Sciences Section, Negaunee Integrative Research Center Field Museum of Natural History Chicago Illinois USA; ^3^ Sciences de la Vie et de l'Environnement, Faculté des Sciences, de Technologies et de l'Environnement Université de Mahajanga Mahajanga Madagascar; ^4^ Zoologie et Biodiversité Animale, Faculté des Sciences Université d'Antananarivo Antananarivo Madagascar; ^5^ Virginia Museum of Natural History Martinsville Virginia USA

**Keywords:** Madagascar, population formation, Pseudoxyrhophiidae, snakes, speciation rates, species persistence

## Abstract

Speciation rates vary substantially across the tree of life. These rates should be linked to the rate at which population structure forms if a continuum between micro and macroevolutionary patterns exists. Previous studies examining the link between speciation rates and the degree of population formation in clades have been shown to be either correlated or uncorrelated depending on the group, but no study has yet examined the relationship between speciation rates and population structure in a young group that is constrained spatially to a single‐island system. We examine this correlation in 109 gemsnakes (Pseudoxyrhophiidae) endemic to Madagascar and originating in the early Miocene, which helps control for extinction variation across time and space. We find no relationship between rates of speciation and the formation rates of population structure over space in 33 species of gemsnakes. Rates of speciation show low variation, yet population structure varies widely across species, indicating that speciation rates and population structure are disconnected. We suspect this is largely due to the persistence of some lineages not susceptible to extinction. Importantly, we discuss how delimiting populations versus species may contribute to problems understanding the continuum between shallow and deep evolutionary processes.

## Graphical abstract

Rates of speciation should be linked to the rate at which population structure forms if a continuum between micro and macroevolutionary processes exists. We find no relationship between rates of speciation and the formation rates of population structure in the Malagasy gemsnakes likely due to differences in species persistence and undetectable extinction among groups.

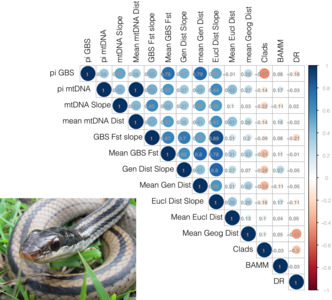

## 1 INTRODUCTION

Speciation rates across the tree of life vary extensively through time (Maliet et al., 2019; Scholl & Wiens, 2016; Sepkoski Jr., 1998; Tietje et al., 2022). Some groups experience early bursts of diversification with subsequent slowdowns, others experience long fuses with recent bursts of speciation, and yet some clades show little change in rate of speciation through time (Burbrink et al., 2012; Diaz et al., 2019; Moen & Morlon, 2014; Springer et al., 2019). Rates of extinction likewise vary through time (Brocklehurst et al., 2015; Ceballos et al., 2020). These differences in rates of speciation and extinction account for why some extant groups like Squamata (*n* = 11,430) have thousands of extant species, whereas their sister taxon Rhynchocephalia only has a single extant species (Vitt & Caldwell, 2009). Despite potential issues with methodology (Louca & Pennell, 2020), the history of speciation rate variation has been confirmed using molecular phylogenies and from the fossil record (Maliet et al., 2019; Pyron & Burbrink, 2013; Sepkoski Jr., 1998).

If the isolation of populations leads directly to the formation of species, then the origins of species and subsequent changes in rates of speciation should be related to the rates at which population structure forms. Given this pattern, taxa with traits limiting genetic connectivity should also produce greater population structure (Burbrink & Ruane, 2021; Rabosky, 2016; Singhal et al., 2022; Vines & Schluter, 2006; Zink, 2014). The lack of genetic connectivity can be initiated by environmental and landscape changes, dispersal ability, or the strength of selection (Harvey et al., 2020; Price et al., 2014; Pyron & Burbrink, 2010; Smith et al., 2014). Therefore, there should exist a relationship between speciation rates and the rate of population formation within these species (Harvey et al., 2017). For example, species groups with higher rates of speciation should then have greater population structure within species if the causes of the formation of lineages remain the same for populations and species. However, if the processes by which populations form are unrelated to the formation of species then population differentiation and rates of diversification should be uncorrelated. Of course, how scientists differentiate populations and species for enumeration of these rates may introduce additional complications (Burbrink et al., 2022; O'Hara, 1993).

There are several patterns of correlation expected between population structure over space and the rates of speciation estimated from trees. A positive correlation between speciation rate and population structure should hold if population structure and subsequent formation of species persists to the present (assuming isolation of populations directly leads to the formation of species). A negative correlation would suggest that high rates of speciation are associated with little population structure, suggesting that clades with high speciation rate contain young species that simply have not had time to form strong population structure. This might be the case given that metrics for estimating tip speciation rates often show higher rates of speciation in groups with many young species (Title & Rabosky, 2019). A lack of correlation indicates that speciation rates and population structure are unrelated, and this can occur for multiple reasons. If populations and species form and become extinct rapidly, then standing diversity and population structure will provide a poor estimate of the actual rate of speciation (Cutter & Gray, 2016; Mayr, 1963; Rosenblum et al., 2012). Of course, this would extend to any population structure not present along the branches of the phylogeny and thus remain unsampled when speciation rates are estimated. This should be the case where populations/lineages not only form readily but also go extinct rapidly (Crampton et al., 2020). Blurring of how populations differ from species may also create some difficulties in assessing if terminal taxa used for estimating speciation rates actually represent species and how these terminals differ from discoverable populations. However, if no correlation is found in scenarios where speciation rates show little variance and the population structure varies widely, this provides a strong conclusion that speciation rates are unrelated to population structure regardless of how extensively population structure variation has been investigated for the presence of distinct terminal lineages.

Previous research that examined squamates in the Cerrado of Brazil (Singhal et al., 2022) and in Australia (Singhal et al., 2018) failed to find a link between speciation rates and population structure. These authors concluded that decoupled speciation rates and demographic population structure might indicate that persistence of population structure and full reproductive isolation are limiting constraints for linking these macro and microevolutionary processes. In contrast, Harvey et al. (2017) found a positive correlation between population differentiation and species diversification in New World Birds. Although there is not yet a consensus, these studies represent some of the only research linking population structure and species rates among diverse clades in large areas.

Here, using a monophyletic group comprising 109 species of Malagasy gemsnakes (Pseudoxyrhophiidae), we examine the correlation between speciation rates estimated over the 23 million years of evolution and extant population structure for 33 species. The Malagasy gemsnakes represent an ecologically diverse group of squamates rivaling the ecological and morphological diversity found in their older continental relatives (Burbrink et al., 2019; Glaw & Vences, 2007). One advantage of investigating gemsnake diversification is that the rates of speciation are confined to a single, relatively young (~23 my), monophyletic group occupying a single island. This reduces complexity introduced by diversification due to multiple colonizations from disparate and older groups and provides a spatially constrained island‐based scenario for testing if a link between speciation and population diversification exists in a young group.

## 2 METHODS

### 2.1 Phylogenetic data

We used the trees inferred from 371 anchored hybrid enrichment (AHE) loci representing 109 species of Malagasy gemsnakes (Pseudoxyrhophiidae; Burbrink et al., 2019). Trees estimated using species tree methods sampled 93% of taxa and were dated by fitting genomic data back to the Astral topology and inferred using TreePL with five fossil dates used across colubrid snakes and cross‐validating the smoothing parameter (Burbrink et al., 2019).

### 2.2 Sampling details for population data

A total of 310 tissue samples were obtained throughout the ranges for 23 previously described snake species found across the island. We note that the use of nominate species names does not indicate that we disagree with previous species delimitation analyses (Burbrink et al., 2019), but rather allows us to assess population structure and admixture from dense population sampling. Consistent with the delimitations from Burbrink et al. (2019), our sampling for this study was equivalent to 33 species (both described and undescribed but recognized as distinct). Tissue samples (muscle, shed skin, or scale clippings, *N* = 126) were collected across Madagascar from 2013 to 2017 and stored in 100% ethanol to preserve DNA. Additional samples (*N* = 184) held in collections were used to complement our recently collected field data (Ambrose Monell Cryo Collection, AMCC; American Museum of Natural History, AMNH; University of Michigan Museum of Zoology, UMMZ; Museum of Vertebrate Zoology, MVZ; and the Université d'Antananarivo, Département de Biologie Animale, UADBA). The number of samples per previously recognized species ranged from 3 to 26 ($\bar{x}$ = 9).

### 2.3 SNP assembly and filtering

**FIGURE 1:**
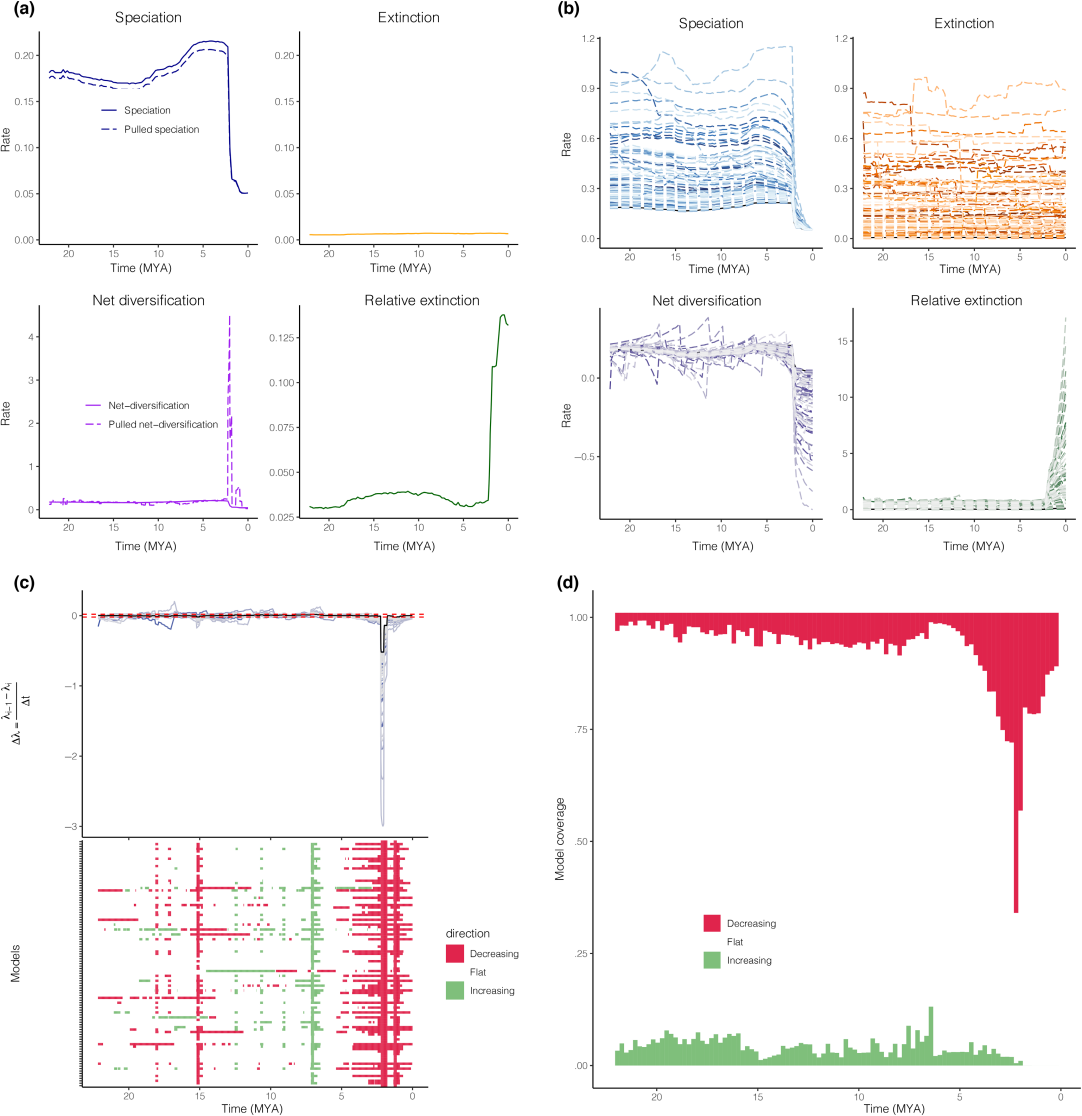
From the tree of gemsnakes: (a) pulled and estimated speciation rates, net diversification and pulled net diversification rates, and extinction and relative extinction rates; (b) estimation of speciation rates, diversification rates, and relative extinction rates from congruence classes; (c) median models over 100 simulations indicating if speciation rate is increasing, decreasing, or flat at each time interval; and (d) posterior probability over time for increasing, decreasing, and flat speciation rates. Panels (a–d) show results for Model 1, where autocorrelated extinction rates were modified (for all Models 1–11, see Figures [Link], [Link]).

**FIGURE 2:**
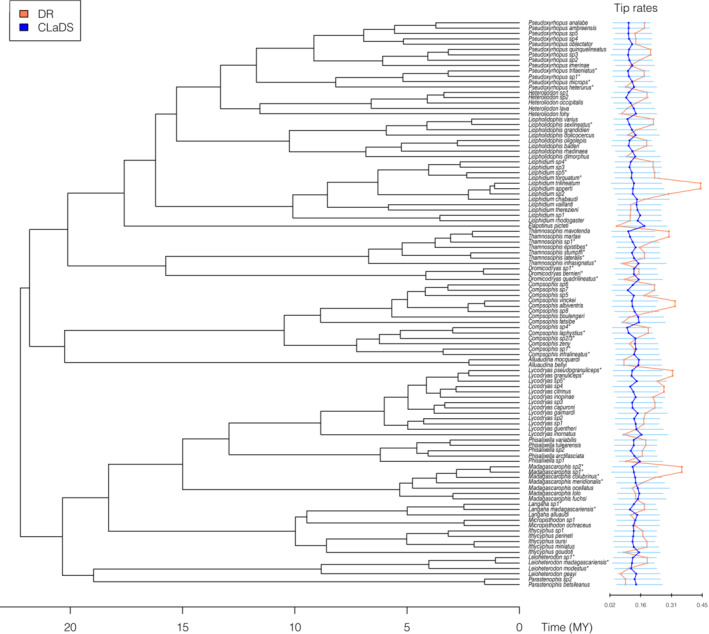
Dated phylogeny of gemsnakes using AHE loci (see Burbrink et al., 2019) and estimates of DR and CLaDS rates of tip speciation.

Genomic DNA was extracted using the DNeasy Blood and Tissue Kit (Qiagen) following the manufacturer's instructions for the following 33 taxa representing 58% of the genera on the tree of gemsnakes (Figures 1 and 2): *Compsophis infralineatus*, *C. laphystius*, *C*. sp.*1*, *C*. sp.2/3, *C*. sp.4, *Dromicodryas bernieri*, *D. quadrilineatus*, *D*. sp.*1*, *Langaha madagascariensis*, *La*. sp.*1*., *Leioheterodon madagascariensis*, *Le. modestus*, *Le*. sp.*1*, *Liophidium torquatum*, *Li*. sp.*4*, *Li*. sp.*5*, *Liopholidophis sexlineatus*, *Lycodryas granuliceps*, *Ly. pseudogranuliceps*, *Ly*. sp.*5*, *Madagascarophis colubrinus*, *M. meridionalis*, *M*. sp.*1*, *M*. sp.*2*, *Pseudoxyrhopus heterurus*, *P. microps*, *P. tritaeniatus*, *P. sp1*., *Thamnosophis epistibes*, *T. infrasignatus*, *T. lateralis*, *T. stumpffi*, and *T*. sp.*1*. We note that GBS data did not recover population structure for *C*. sp.2 and sp.3 as identified in Burbrink et al. (2019); therefore, these two groups were collapsed here into *C*. sp.2/3.

Samples were sent out for genotyping‐by‐sequencing (GBS; Elshire et al., 2011) at the Cornell Institute of Genomic Diversity. Genomic DNA was digested with the enzyme PstI (recognition sequence: CTGCAG) and sample‐specific barcoded adapters as well as a common adapter were ligated to the sticky end of the fragments. Samples were purified, pooled for a size selection PCR, and then purified a second time. Libraries were sequenced on an Illumina HiSeq 2000 at the Cornell Core Lab Center.

We processed paired‐end Illumina reads using the bioinformatics pipeline ipyrad v0.5.13 (Eaton & Overcast, 2020; additional details can be found in Text S1). Raw SNP files were filtered using vcftools v0.1.13 (Danecek et al., 2011) to test different filtering schemes to maximize the number of loci retained while maintaining the patterns of differentiation between populations found by more conservative filtering approaches (min depth = 10; removing sites with >15% of missing data across all individuals). We compared results for different values of the missing data threshold and found that 20%–60% yielded similar trends; therefore, we used the 20% value that retained more SNPs.

### 2.4 mtDNA

We also used COI mtDNA data for each of these species (numbers per species 2–26, $\bar{x}$ = 8) sequenced in Burbrink et al. (2019) to understand whether population structure when related to rates of speciation using mtDNA was similar to the nuclear genomic data generated here. Using mtDNA also helped determine if this locus provides adequate information about population structure.

### 2.5 Speciation rates

A previous study found that rates of diversification in the gemsnakes declined significantly over the entire tree during the Pleistocene (Kuhn et al., 2022). Since then, Louca and Pennell (2020) demonstrated that rates of speciation estimated from molecular phylogenies may be congruent with a wide range of diversification scenarios. To examine whether the rates in gemsnakes were declining and also estimate rates of diversification for each species, we fit the estimated trees to a number of congruent scenarios using the R package CRABS (Kopperud et al., 2023). We first estimated the pulled speciation rates from our observed tree (Helmstetter et al., 2022), which provided values that differed from empirical rates due to missing taxa or extinction. From this, we constructed a series of rate congruence classes based on our empirical data. We divided these into two broad classes, estimating changing speciation and changing extinction rates. For the former, we modeled extinction and inferred speciation and vice versa for the latter. We tested temporally autocorrelated extinction rates (Model 1) and linearly and exponentially increasing extinction rates (Models 2–6). We then used the same models but for varying speciation rates while estimating extinction rates (Models 7–10). Finally, we tested for episodic extinction rates with stochastic noise (Model 11). As suggested by the authors (Kopperud et al., 2023) and in the CRABS documentation (https://github.com/afmagee/CRABS), to estimate alternative extinction or speciation rate changes we used the Gaussian Markov random field (GMRF) model or horseshoe Markov random field (HSMRF) model (for autocorrelated rates) to generate extinction or speciation rates depending on the congruence class model. For all 11 congruence models, we generated 100 estimates per model. We used a 0.02 threshold of detection in change of speciation or extinction rate over 100 time slices since the origin of the gemsnakes (~23 my). For all increases or decreases in extinction, we used a twofold change with a maximum rate of extinction = 1.0. Similarly, we used a twofold change for speciation rates with a maximum change at 0.5; we note that values above this failed to generate simulations.

We calculated tip rates of speciation and correlated these to metrics estimated using population structure with two model‐based approaches, CLaDS and BAMM, and one semiparametric approach, the inverse splits statistic (DR). The CLaDS method uses Bayesian inference to estimate diversification rates for each branch on the tree and models changes in diversification rate at every speciation event (Maliet et al., 2019) requiring only the dated tree and fraction of species present (93%). CLaDS was run in Julia (Bezanson et al., 2017) with three MCMC chains stopping when Gelman statistics (Gelman et al., 2013) were below 1.05 as recommended by the authors (Maliet et al., 2019). BAMM uses an rjMCMC approach to explore models of diversification to detect heterogeneity in evolutionary rates (Rabosky et al., 2014; Rabosky, 2014; see Burbrink et al., 2019, for details on running BAMM with this dataset). For both of these methods, we pulled recent speciation rates (tip rates) as featured in each program. The DR statistic represents a weighted mean of inverse branch lengths from the tip to the root of the tree (Jetz et al., 2012; Title & Rabosky, 2019) and provides speciation rate estimates at the tip of the tree. To better understand how these tip rates related to other snakes with similar ages of origin that are also monophyletic by region, we used CLaDS and DR to estimate diversification rates using previously published phylogenies representing the New World ratsnakes (Lampropeltini, ~24 myo; Chen et al., 2017), the New World watersnakes (Thamnophiini, ~18 myo; McVay et al., 2015), and the New World pitvipers (Crotalinae, ~25 myo; Alencar et al., 2016). These trees represented 95%, 86%, and 66% of extant species, respectively (Uetz et al., 2009). We examined the range of variation on tip rates among all four groups.

### 2.6 Population structure over space

To examine population structure over space, we estimated isolation by distance (IBD) using both the GBS data and mtDNA. Following Singhal et al. (2022), we estimated Fst between individuals using the R package BEDASSLE (Bradburd et al., 2013; R Core Team, 2010), calculated *F*_st_/1 − 1*F*_st_ and regressed this against log (geographic distance), and took the slope (β_IBD_) to represent population structure over space. Because *F*_st_ is generally estimated using populations, we also calculated nucleotide diversity (*π*) for each species. Additionally, we also estimated genetic distance in Adegenet (Jombart, 2008) and Euclidean distance and took the slope from these two measures regressed against log (geographic distance). We similarly calculated these last two measures (genetic and Euclidean distance regressed against the log of geographic distance) for mtDNA data and examined the correlation between mtDNA and GBS estimates. Finally, all measures of slope (population structure over space) were correlated with CLaDS, BAMM, and DR estimates of tip speciation rate.

We also used phylogenetic least squares to examine the relationship between population genetic metrics and speciation rate estimates. To reduce problems with the assessment of multiple phylogenetic regression coefficients, we eliminated multicollinearity among these predictor variables using the vif function in the R package car (Fox & Weisberg, 2018). Here, we subsampled variables until our final set retained variable inflation factors below 10, leaving us with β_IBD,_ mean mtDNA distance, the slope of genetic distance, the slope of Euclidian distance, and *π* for GBS data. We then assessed phylogenetic signal for each of the three estimates of tip speciation for values of lambda (Pagel, 1999) and Blomberg's *K* (Blomberg et al., 2003) with 999 replicates in the function phylosig in the caper package (Orme, 2018). We examined the relationship between the reduced population metric data on estimates of tip speciation rates using the function pgls in caper with a maximum likelihood estimate for the lambda transformation.

## 3 RESULTS

### 3.1 Speciation rates

Our estimation of pulled and empirical diversification rates showed a downturn in speciation during the Pleistocene (Figure 1). Congruence datasets where extinction is modified as temporally autocorrelated, linearly and exponentially increasing and decreasing (Models 1–5), and episodic (Model 11) all showed a downturn in speciation rates in the Pleistocene over all 100 replicates for each model (Figure 1, and Figures [Link], [Link]). Where speciation was modeled as temporally autocorrelated, linearly, and exponentially increasing or decreasing, we found that estimates of extinction rates remained generally unchanging over most of the replicates per model (see Figures [Link], [Link]).

**FIGURE 3:**
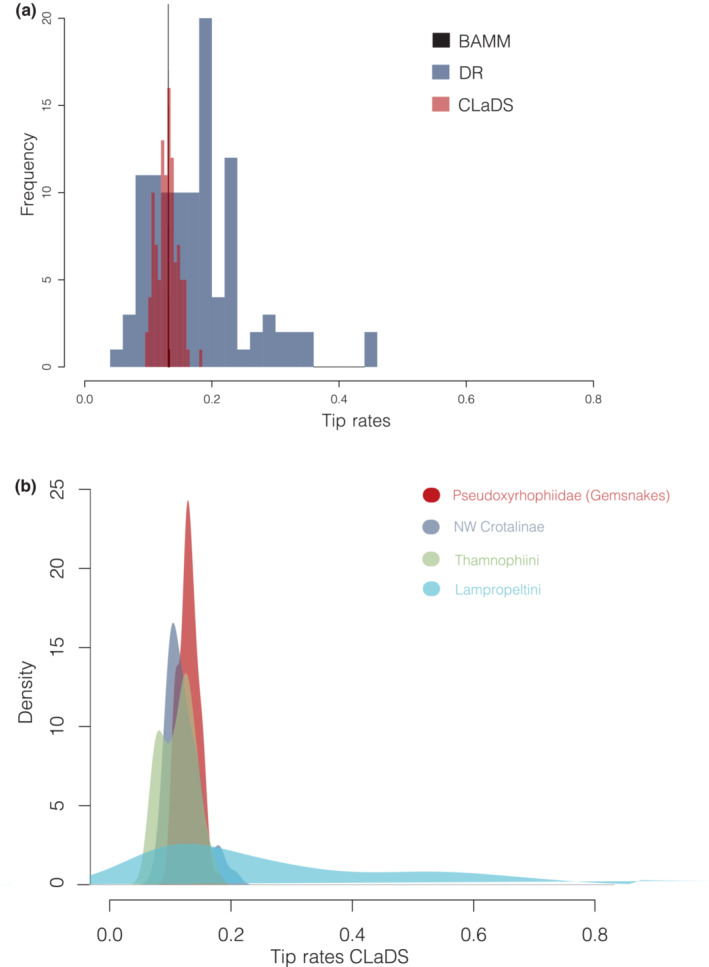
(a) Estimates of tip speciation rates from BAMM, DR, and CLaDS for Pseudoxyrhophiidae (gemsnakes) and (b) estimates of tip speciation rates from CLaDS for Pseudoxyrhophiidae (gemsnakes), New World Crotalinae, Thamnophiini, and Lampropeltini.

**FIGURE 4:**
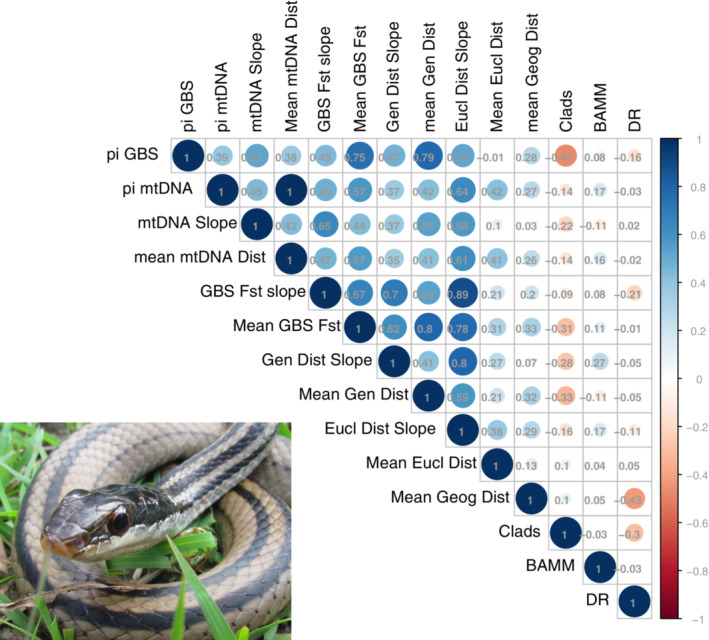
Correlation among various measures of population structure for genomic (GBS) and mtDNA data, including pi, slope (genetic variation over space estimated as either Fst, genetic distance, or Euclidean distance), mean geographic distance among points, and estimates of tip speciation rate from CLaDS, BAMM, and DR. Correlation coefficients are displayed, and intensity of color represents greater positive (blue) or negative correlation (orange). Photograph of *Dromicodryas quadrilineatus* by Frank Burbrink.

Estimation of tip speciation rates varied widely across methods (Figure 3). Variance in rate estimation increased from BAMM to CLaDS to DR. Interestingly, CLaDS and DR were negatively correlated (ρ = −.46, *p* < 7.38 × 10^−7^), where short branches have higher rates as estimated by DR and CLaDS had higher rates for taxa with long branch lengths (Figure 4). The modeling approach in CLaDS, where the gemsnakes showed declining speciation rates (Figure 1) toward the present, estimated a decrease in speciation rates for tips with shorter lengths. For trees with little rate variation, most changes were concentrated at internodes showing a decline in overall speciation rates towards the present (O. Maliet, personal communication). This is the opposite pattern predicted by the semiparametric method, DR, where rate information is not modeled over the entire tree and therefore short terminal branches with more branching points between the tip and root reflect higher speciation rates. The Lampropeltini, New World Crotalinae, and Thamnophiini all had positively and significantly correlated (ρ) CLaDS and DR rates, .93 (*p* < 2.2 × 10^−16^), .46 (*p* < 1.28 × 10^−7^), and .36 (*p* < .008). All of these groups showed greater variability in tip speciation rates than the gemsnakes (Figure 3). The coefficient of variation for CLaDS estimates of tip rates was larger for the New World Crotalinae (0.23) and Thamnophiini (0.24) when compared to the gemsnakes (0.13) and much larger for Lampropeltini (0.73).

### 3.2 Population structure and speciation rates

Significant population structure was present in all species (GBS x̄_*F*st dist_ = 0.44, SD = 0.11). Both mtDNA and GBS genetic distances and slopes were significantly correlated (ρ = .414, *p* = .015; ρ = .371, *p* = .03, respectively) reflecting that both genomes were tracking the same population structure. Most species had a significant positive slope (*p* < .05) between geographic distance and genetic distance (mtDNA; 65%) or *F*_st_/1 − 1*F*_st_ (GBS; 74%). For GBS, all measures of slope using *F*_st_, genetic distance, and Euclidean distance were significantly correlated (ρ = .70–.88; *p* < 3.76 × 10^−6^; Figure 4).

**FIGURE 5:**
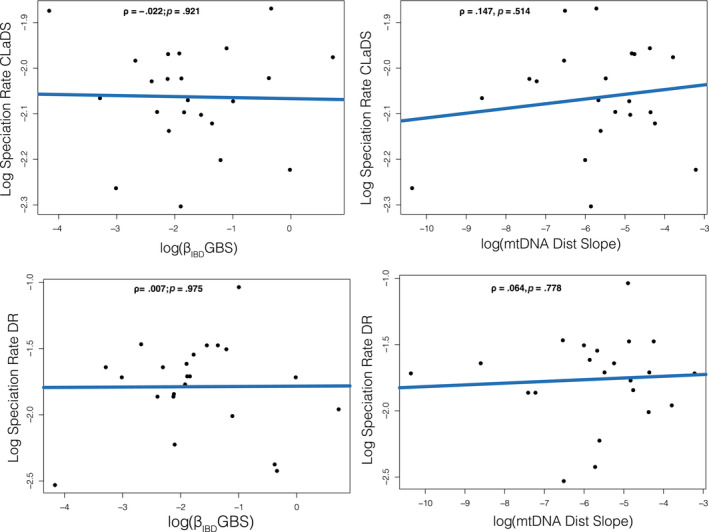
Scatterplots showing the relationship between log slope (β_IBD_ for GBS and genetic distance over space for mtDNA) for log speciation rate estimates from CLaDS and DR for taxa with more than four samples.

No measures of slope (β_IBD_; genetic distance/*F*_st_/1 − 1*F*_st_) from mtDNA or GBS regressed against geographic distance were significantly correlated with tip measures of speciation from CLaDS, BAMM, or DR (*p* = .112–.90; Figures 4 and 5) even when restricting the minimum number of samples to be greater than 10. Similarly, measures of genetic distance and Euclidean distance were not significantly correlated with any of the tip measurements of speciation. The mtDNA estimates of π were not significantly correlated with any of the measurements of speciation at the tips; however, π using the GBS data was significantly correlated with tip speciation rate estimates from CLaDS (ρ = −.47, *p* = .005), suggesting that nucleotide diversity was higher in those taxa with lower rates of speciation. Phylogenetic signal was only significant for CLaDS using Blomberg's *K* (*K* = 0.395; *p* = .006) and for DR for both lambda (λ = 1.050, *p* = 1.21 × 10^−5^) and for Blomberg's *K* (*K* = 0.600, *p* = .001). When accounting for phylogeny and multicollinearity among population genetic metrics, we found that no models were significant for CLaDS (adjusted *r* ^2^ = .090, *p* = .349), BAMM (adjusted *r* ^2^ = .058; *p* = .404), or DR (adjusted *r* ^2^ = −.279, *p* = .886).

## 4 DISCUSSION

We find no association between the formation of population structure over space and speciation rates in the gemsnakes of Madagascar. This holds for mtDNA (COI) and nuclear genomes (Figure 4). Considering congruent patterns across different diversification scenarios, based on pulled diversification rates (Figure 1), we confidently show that rates of speciation are declining in these snakes. The rate of decline in the Pleistocene and related tip speciation rates is unlinked to the extent of population structure in these snakes.

Our results are similar to studies on squamates in Brazil and Australia (Singhal et al., 2018, 2022) that also fail to show a link between speciation and population divergence over space. The gemsnakes have less variation in rates of speciation over all terminal taxa than other groups of colubrid snakes that are monophyletic by region that also have a similar age of origin (Figure 3). This shows that there is no link between the degree of population structure over space and speciation rates in gemsnakes; tip‐estimated speciation rates have little variation despite population structure varying massively for those same terminal species (Figure 5). We also note that tip rates of speciation from our selection of terminal species for examining population structure account for 91.5% of the range of all speciation rate estimates. These results are in contrast to studies on birds that showed a positive correlation between speciation rates and population divergence or the formation of subspecies (Harvey et al., 2017; Haskell & Adhikari, 2009; Phillimore, 2010). It is unclear yet whether there is a general trend, but all squamates so far examined fail to show this link. The aforementioned studies on squamates sampled a much deeper group of taxa using population genome data (target‐capture loci) and a phylogeny of all squamate species driven mainly by mtDNA data including imputed data (Tonini et al., 2016) also failed to find a link. In the gemsnakes, this lack of correlation indicates that populations form or go extinct at variable rates despite declining rates of speciation throughout the Pleistocene. If this general relationship holds, it suggests the processes that form populations are unrelated to the persistence of species in squamates, but not in birds. To note, the bird study (Harvey et al., 2017) investigated 173 taxa and used mtDNA data for estimating both the phylogenetic tree and population structure, so it is unclear whether this relationship is valid for a more diverse estimate of the genome. We note that our mtDNA and nuclear genomic data results are largely correlated.

Previous studies (Harvey et al., 2017; Singhal et al., 2022) showed high variation in the estimation of speciation rates across a more diverse sampling of taxa. Depending on the measure, CLaDS and BAMM show much less variation (SD = 0.016) around speciation rates than the DR method (SD = 0.085; Figure 3), though no methods are significantly correlated with population structure. The former two methods model rates of speciation using the entire tree structure and therefore may be detecting overall trends better than the DR method. In a study comparing methods for estimating speciation rates at tips, it was shown that BAMM is more accurate than DR, particularly given extinction and changes in rates (Title & Rabosky, 2019); however, no comparisons between ClaDS and any methods for estimating tip rates exist. If modeling‐based methods are preferred given that they account for rate changes over the entire tree, such as ClaDS, then this indicates that rates of speciation are similar despite large variance on population structure. These patterns are similar with BAMM.

Even though isolation by distance and isolation by ecology (IBE) are often considered the first steps for the formation of species (Arnold & Fristrup, 1982; Avise et al., 1987; Baptestini et al., 2013; Jablonski, 2008; Rundle & Nosil, 2005), resulting patterns of diversification may be later offset and eroded by population/species persistence. Therefore, the continuum between microevolutionary processes and macroevolutionary patterns may be disrupted due to variance in the persistence of populations, a necessary step to be considered as distinct lineages. This variance may be associated with extinction by forces such as environmental change, competition, disease, or hybridization (Burbrink et al., 2022; Raia et al., 2016; Rhymer & Simberloff, 1996; Vonlanthen et al., 2012). It could be argued that additional processes are relevant to prevent extinction via hybridization, such as increasing genomic incompatibilities thus enhancing reproductive isolation (Mayr, 1963; Wolf & Ellegren, 2017; Wu, 2001). Our results therefore indicate that the formation of population structure is not a rate‐limited step for the formation of species but rather may be offset and disrupted by rapid extinction. That is, if the isolation of populations directly leads to the formation of species.

Most species of gemsnakes investigated here show population structure over space, though three taxa have larger than average range sizes with little genetic variation (and thus probably represent recent widespread expansion). Therefore, while the estimated rates of speciation and population differentiation are disconnected, there is still a general trend for extant species to show significant population structure across the island associated with range size. Even though the area of Madagascar and phylogenetic diversity is smaller in comparison with most other studies examining this link, the gemsnakes and other taxa on this island show major phylogeographic structure at the intersection of six starkly contrasting biomes (Burbrink et al., 2019; Kuhn et al., 2022; Raxworthy et al., 2007; Yoder & Heckman, 2006) and species endemism is also apparent in much smaller climatic zones (Pearson & Raxworthy, 2009). Thus, it appears that this structure across these biomes is not directly translated into stable lineages/species over longer periods of time. Additionally, historical demographic dynamics, such as recent widespread expansion possibly associated with Quaternary climate change, are commonly recovered for squamates in Madagascar (Burbrink et al., 2019; Florio et al., 2012; Kuhn et al., 2022; Raxworthy et al., 2007; Yoder & Heckman, 2006) and may also enhance the rate of population formation via IBD or IBE (isolation by ecology) or reduce this rate via hybridization (Delrieu‐Trottin et al., 2017; Marques et al., 2017). We show strong support for decreased speciation rates in the gemsnakes during the Pleistocene, which may have been due to diversity‐dependent diversification processes (or see alternative explanations in Burbrink et al., 2019). Understanding complex population structure and processes responsible for that structure for each species is out of scope of this paper and also constrained by the number of samples available. However, we advocate further exploration of phylogeographic lineage structure within the gemsnakes to better understand genetic diversity within geographically structured populations and relate those speciation rates as well.

One potential limitation for all studies connecting rates of speciation and population formation is the difficulty determining when a population becomes a species. All of the aforementioned studies implement a two‐step process influenced by how taxa are chosen for each step: (1) estimating speciation rates with trees and (2) estimating population structure within taxa. If users cannot quantify how a population differs from a species (Burbrink et al., 2022; Kizirian & Donnelly, 2008), then terminal taxa used to estimate speciation rates may be arbitrarily selected. This could either increase or decrease rates of speciation estimates depending on if more or less taxa are used in that step. Additionally, if all populations in a particular species are actually species but not included in Step 1, then these have been selectively restricted from the speciation rate estimation and kept in Step 2 for estimating population structure. If these populations/lineages are considered species and used in Step 1, then estimates of population structure in Step 2 for the remaining terminals are likely to be reduced. This would suggest that it should generally be unclear whether higher rates of speciation should be positively correlated with increased population structure within species owing to the propensity to speciate, or, alternatively, negatively correlated with decreased population structure following rapid speciation. Furthermore, it is possible that with younger and diverse groups, accurate estimates of speciation could be obtained without an abundance of extinction events and could be better correlated to population structure. If, however, delimitation is performed idiosyncratically, then there should be no link between speciation rates and population formation. Therefore, analyses that address the continuum between population rate formation and speciation rates may be forcing the key units of measure into categories unable to reveal this continuum.

Estimates of speciation rates on trees only yield speciation rates for those taxa which persist over long periods of time and therefore cannot account for extinction of ephemeral species (ghost lineages). These considerations regarding species delimitation and how species differ from population structure in a phylogenetic context should be kept in mind when developing new ways to link the formation of population speciation rates. Perhaps to better understand this continuum, all lineages, regardless if they are species or populations, should be included in phylogenetic analyses of diversification to test for temporal autocorrelation of speciation rates between shallow (population level) and deep nodes. Results that have previously delimited species using coalescent methods (Burbrink et al., 2019) may be free from some of these confounding factors and therefore provide a strong inference that speciation and population formation are uncorrelated. Therefore, understanding the rate at which populations or lineages persist or go extinct through time is key to testing links between microevolutionary processes and macroevolutionary patterns. Future work investigating how species form with regard to genomic structure in the gemsnakes and how this relates to population/lineage persistence is a logical step to better estimating the rate at which species form and are lost via hybridization. Extending this to taxa of varying ages will provide insight into the length of time that lineages, and logically species, persist on Madagascar.

## AUTHOR CONTRIBUTIONS

**Frank T. Burbrink:** Data curation (lead); formal analysis (lead); funding acquisition (equal); investigation (lead); methodology (lead); project administration (lead); supervision (lead); visualization (lead); writing – original draft (lead); writing – review and editing (equal). **Sara Ruane:** Data curation (equal); writing – review and editing (equal). **Nirhy Rabibisoa:** Investigation (equal); resources (equal); writing – review and editing (equal). **Achille P. Raselimanana:** Data curation (equal); investigation (equal); writing – review and editing (equal). **Christopher J. Raxworthy:** Funding acquisition (equal); investigation (equal); writing – review and editing (equal). **Arianna Kuhn:** Conceptualization (equal); data curation (equal); formal analysis (equal); methodology (equal); writing – review and editing (equal).

## CONFLICT OF INTEREST STATEMENT

We have no competing interests.

## Supporting information


Appendix S1:

Appendix S2:

Figure S1:

Figure S2:

Figure S3:

## Data Availability

All supplementary data, which include phylogenetic trees, GBS data, code, and outputs in Data S1–S12 are available on Data Dryad at the following link: https://doi.org/10.5061/dryad.rr4xgxdcf. All raw reads have been uploaded to the Sequence Read Archive (SRA) database under BioProject ID: PRJNA981199.
