# Supplementary material for: Speciation rates are unrelated to the formation of population structure in Malagasy gemsnakes
Source: Ecol Evol. 2023 Jul 28;13(8):e10344. doi: 10.1002/ece3.10344 (PMC10375368; doi:10.1002/ece3.10344)
Supplement: Supplementary file 1 — Appendix S1: [file ECE3-13-e10344-s005.zip › Text_S1.docx]

We processed paired-end Illumina reads using the bioinformatics pipeline ipyrad (v0.5.13, Eaton & Overcast, 2020). Sequences were demultiplexed using their unique barcode sequences. For *de novo* assembly of paired-end GBS loci, bases with a PhredQ score less than 30 were converted to ‘Ns’ and reads with 15 or more uncalled bases were discarded. GBS approaches use common adapters and sequence short fragments from both ends, therefore we identified and trimmed adapter sequences using stringent parameter settings. Reads were searched for the common Illumina adapter, the reverse complement of the second cut site (if present), and the barcode itself (if present), then trimmed reads <35 bp in length were discarded. For basecalling, we allowed for a maximum number of 5 uncalled and 8 heterozygous bases in consensus sequences. For homologous sequence clustering, the threshold was set to 0.85 to account for the presence of Ns, indels, sequencing errors, or polymorphisms. We allowed for a maximum number of 20 SNPs per locus, but also generated assemblies with only one SNP per locus for downstream analyses that are sensitive to bias due to redundant signal from linked SNPs. We generated these two alternate datasets pertaining to the minimum number of samples that must have data at a given locus for it to be retained in the final data set. The first had a minimum of 4, resulting in more loci that were more sparsely sampled (more missing data) to examine patterns of missingness. For the second assembly, we set this minimum to 0.75x (x = total number of samples in each species group assembly) to generate a matrix with fewer loci, but less missing data. When clustering consensus sequences across all samples, each species complex was branched from the original dataset and independently analyzed. The program fastqc was run for sequence quality control. Due to considerable adapter contamination, the adapter filter was set to 2, to perform more stringent filtering, such that reads were searched for the common Illumina adapter, plus the reverse complement of the second cut site (if present), plus the barcode (if present), and this part of the read was trimmed. This filter was applied using code from the software cutadapt, which allows for errors within the adapter sequence (Martin 2011).

Raw SNP files were filtered using vcftools (v0.1.13; Danecek et al., 2011) to test different filtering schemes to maximize the number of loci retained while maintaining the patterns of differentiation between populations found by more conservative filtering approaches (min depth = 10; removing sites with >15% of missing data across all individuals). We compared results for different values of the missing data threshold and found that 20%–60% yielded similar trends; therefore, we used the 20% value that retained more SNPs (Huang & Knowles, 2016). We did a preliminary assessment of population structure and admixture using sNMF (Frichot et al., 2014, see 1.3 below), and identified individuals that showed significant correlation with estimates of spurious population admixture and coverage per locus that also had less than 30% of total locus coverage, and reran step 7 of ipyrad to generate new assemblies with these individuals removed (*n* = 94).

References

1. Eaton DAR, Overcast I. 2020 ipyrad: Interactive assembly and analysis of RADseq datasets. *Bioinformati cs* **36**, 2592–2594.

2. Frichot E, Mathieu F, Trouillon T, Bouchard G, François O. 2014 Fast and efficient estimation of individual ancestry coefficients. *Genetics* **196**, 973–983.

3. Huang H, Knowles LL. 2016 Unforeseen Consequences of Excluding Missing Data from Next-Generation Sequences: Simulation Study of RAD Sequences. *Syst. Biol.* **65**, 357–365.
