# Supplementary material for: Speciation rates are unrelated to the formation of population structure in Malagasy gemsnakes
Source: Ecol Evol. 2023 Jul 28;13(8):e10344. doi: 10.1002/ece3.10344 (PMC10375368; doi:10.1002/ece3.10344)
Supplement: Supplementary file 3 — Figure S1: [file ECE3-13-e10344-s002.pdf]

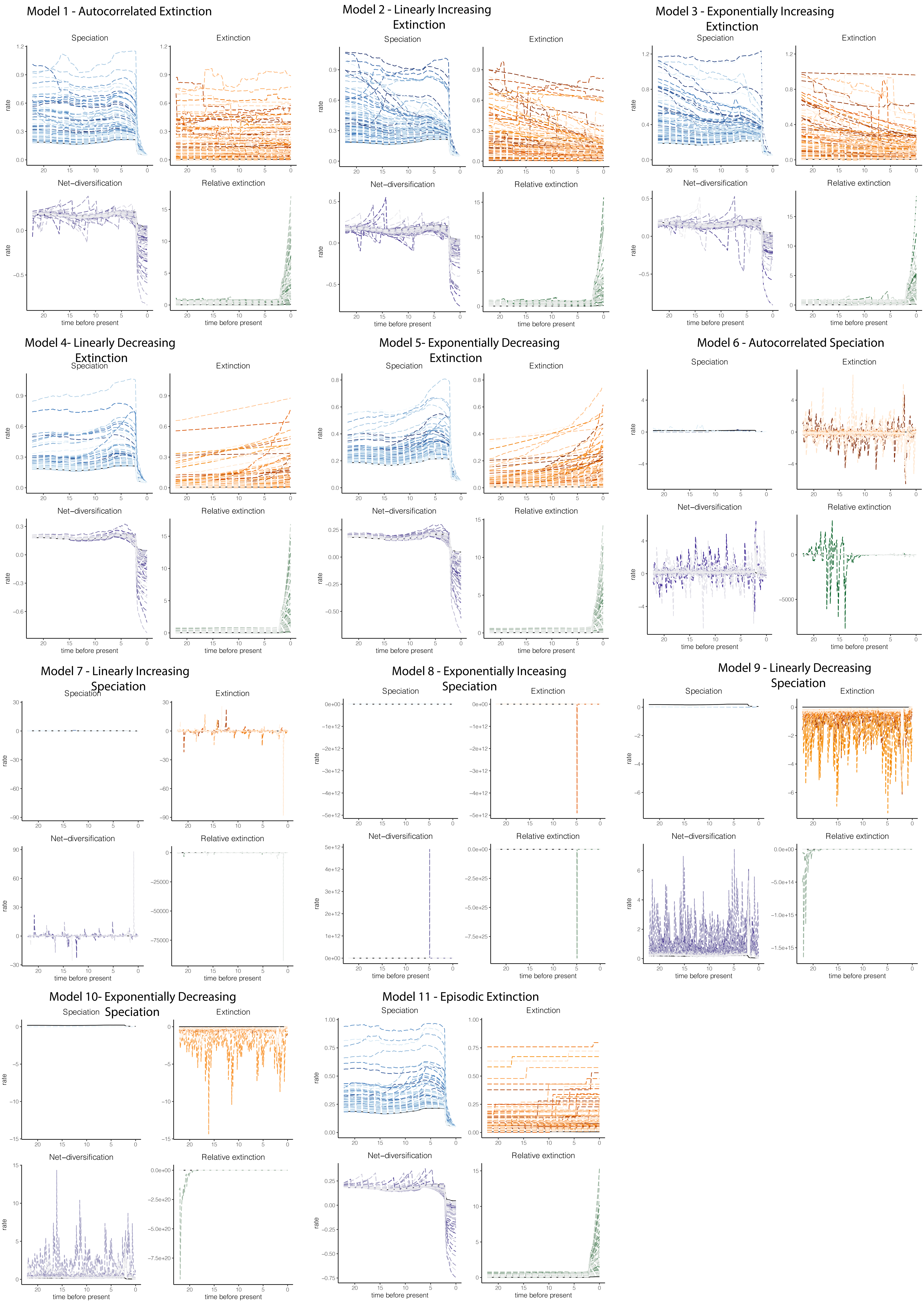

Model 1 - Autocorrelated Extinction
Model 2 - Linearly Increasing Extinction
Model 3 - Exponentially Increasing Extinction
Model 4- Linearly Decreasing Extinction
Model 5- Exponentially Decreasing Extinction
Model 6 - Autocorrelated Speciation
Model 7 - Linearly Increasing Speciation
Model 8 - Exponentially Inceasing Speciation
Model 9 - Linearly Decreasing Speciation
Model 10- Exponentially Decreasing Speciation
Model 11 - Episodic Extinction
Speciation
Extinction
Net−diversification
Relative extinction
rate
time before present
