# Supplementary figures and images for: Speciation rates are unrelated to the formation of population structure in Malagasy gemsnakes
Source: Ecol Evol. 2023 Jul 28;13(8):e10344. doi: 10.1002/ece3.10344 (PMC10375368; doi:10.1002/ece3.10344)

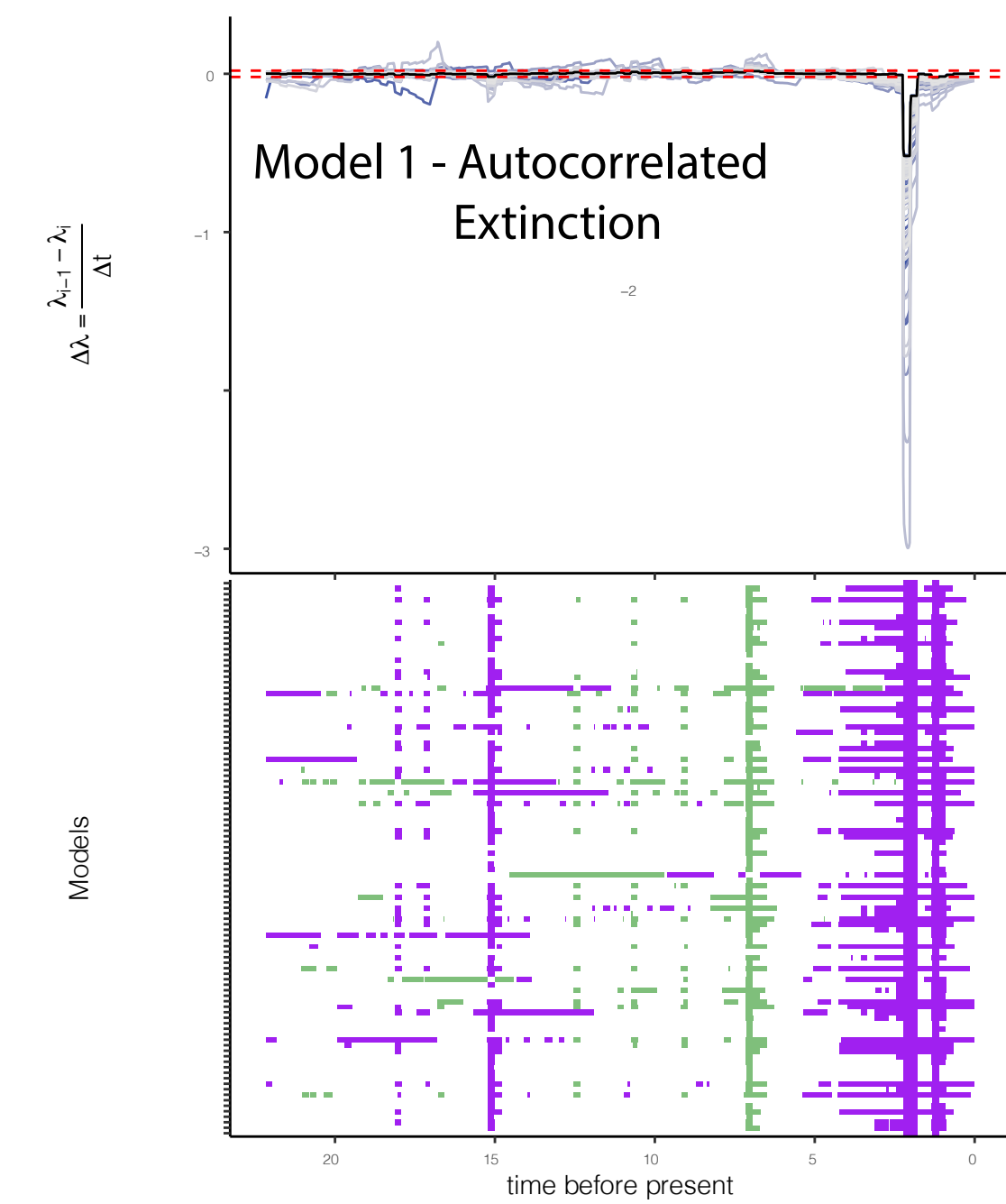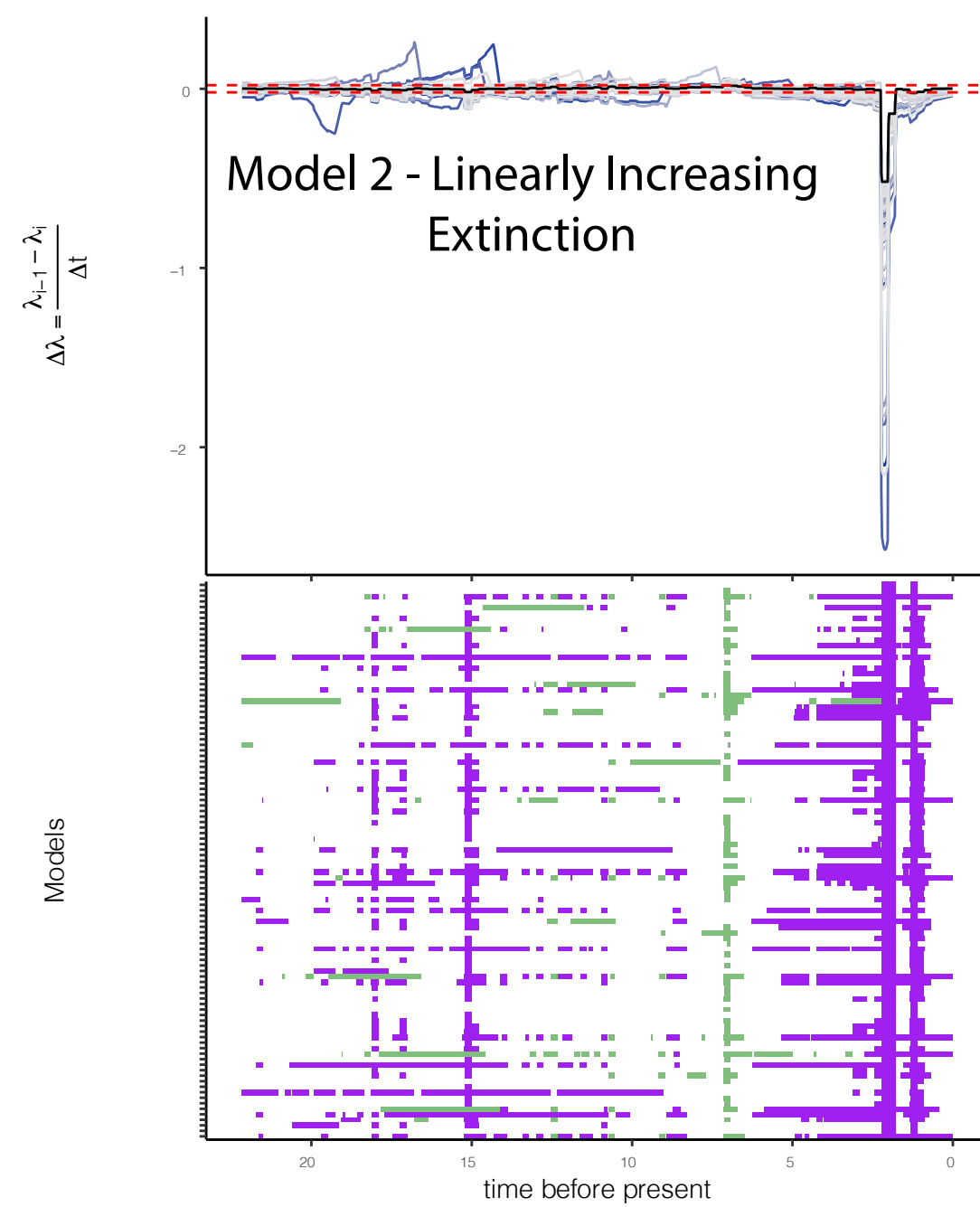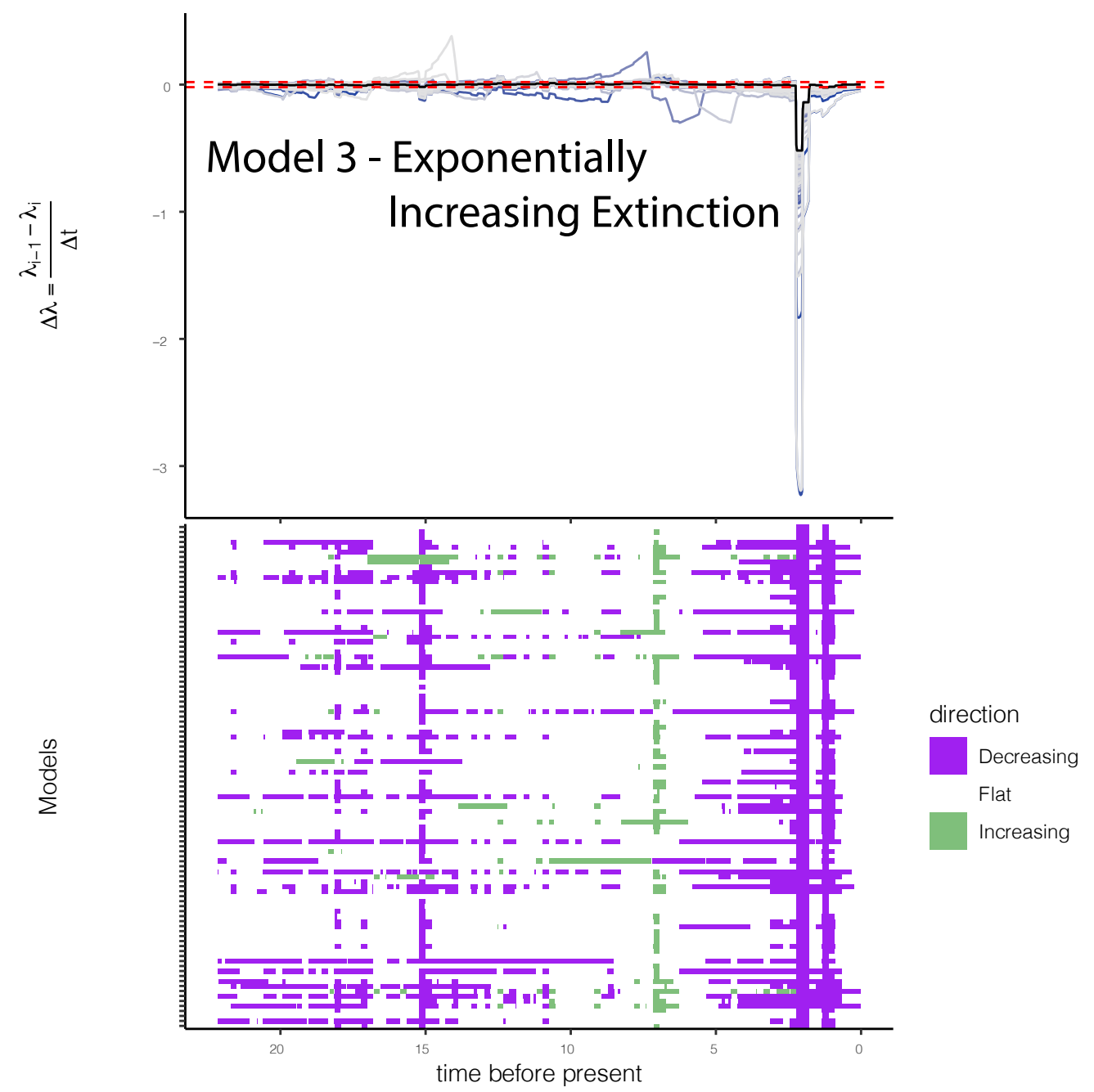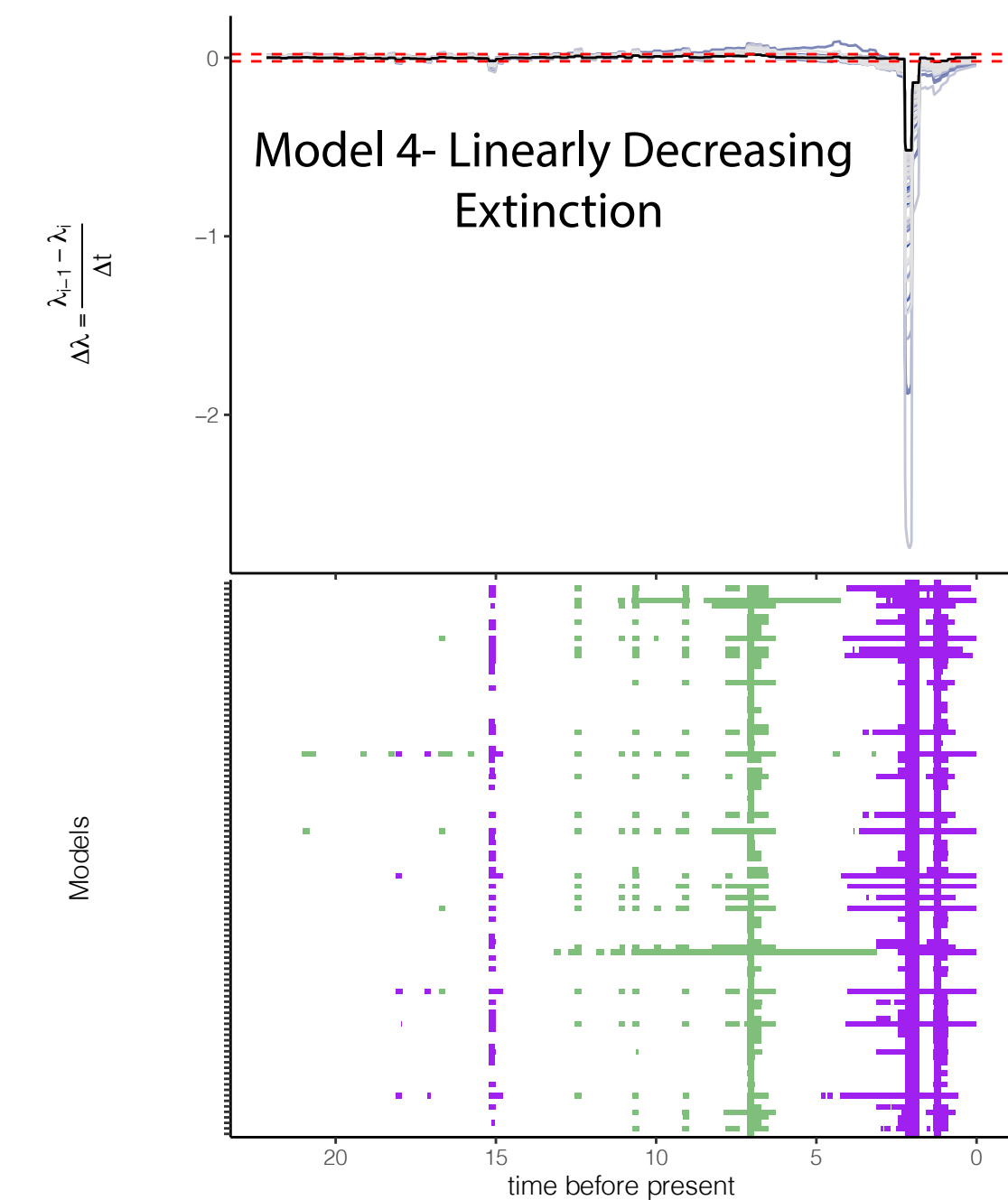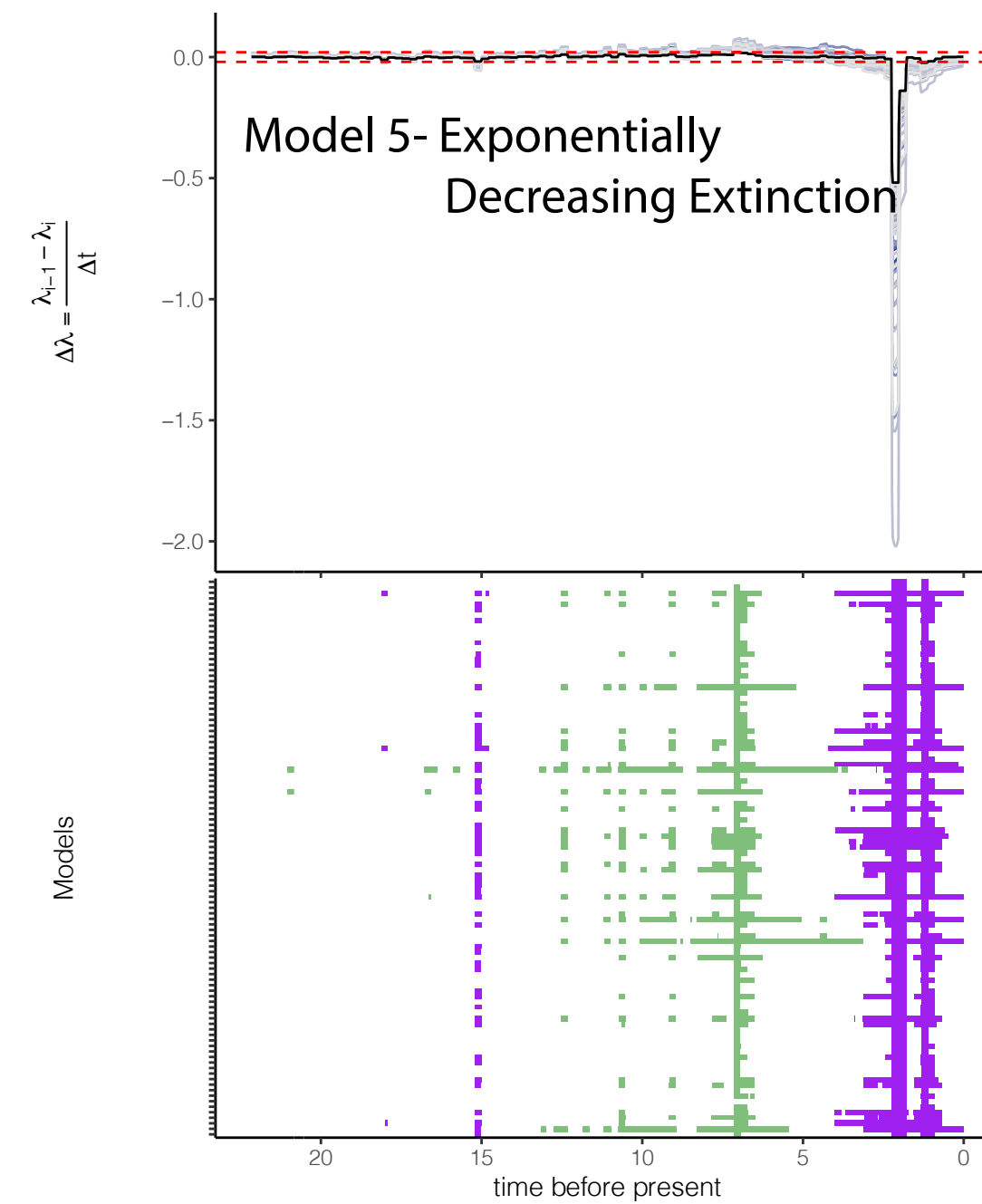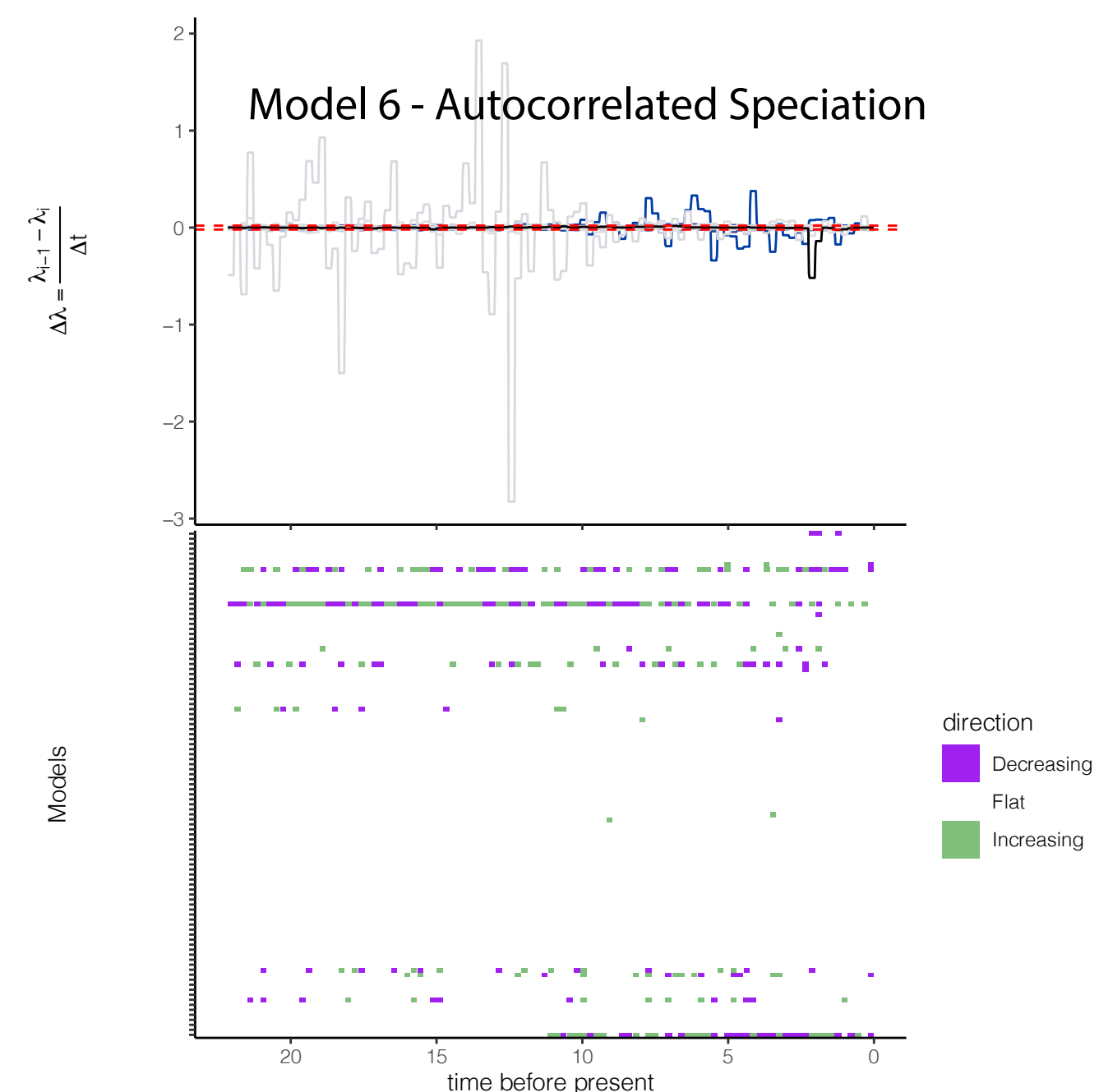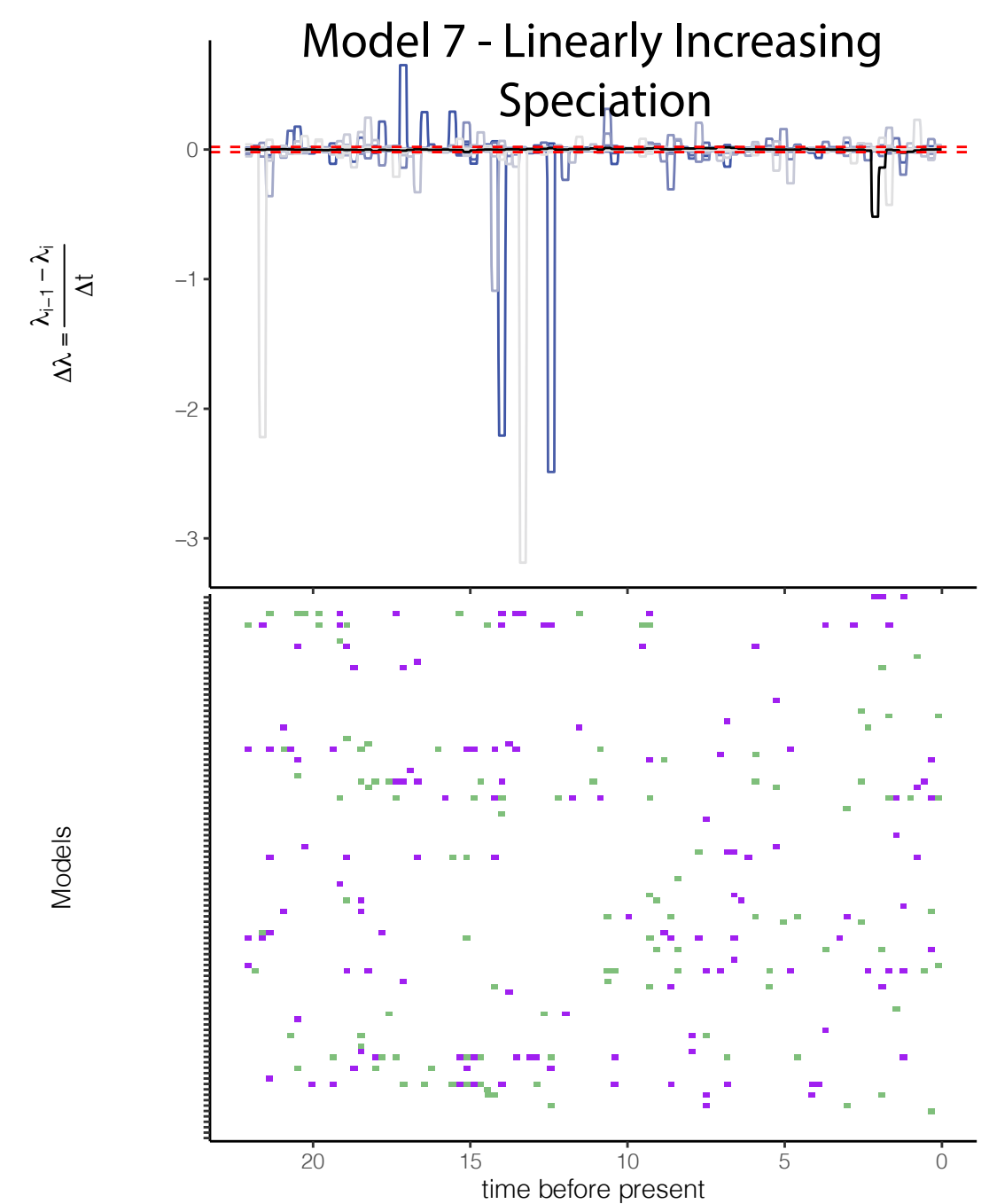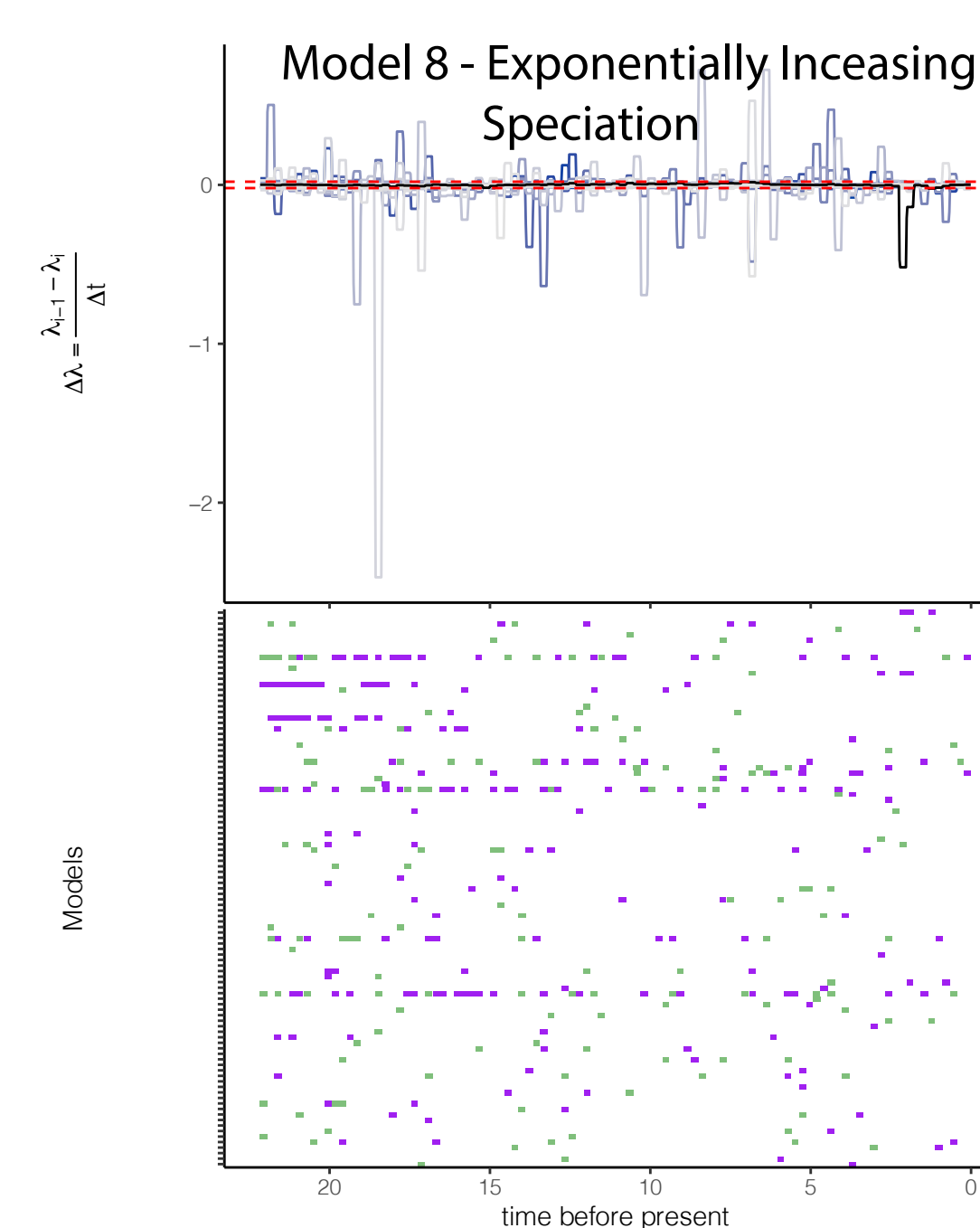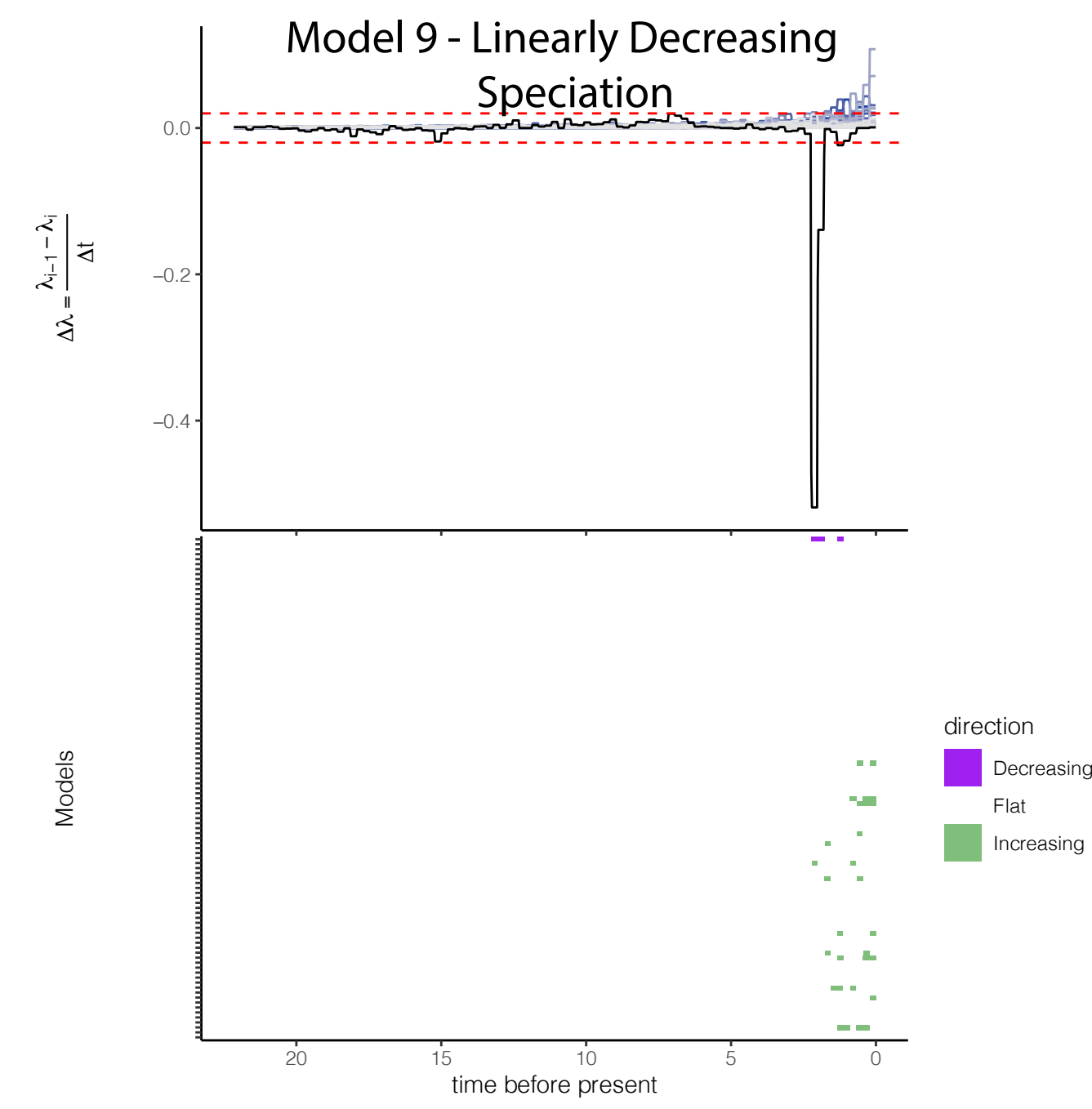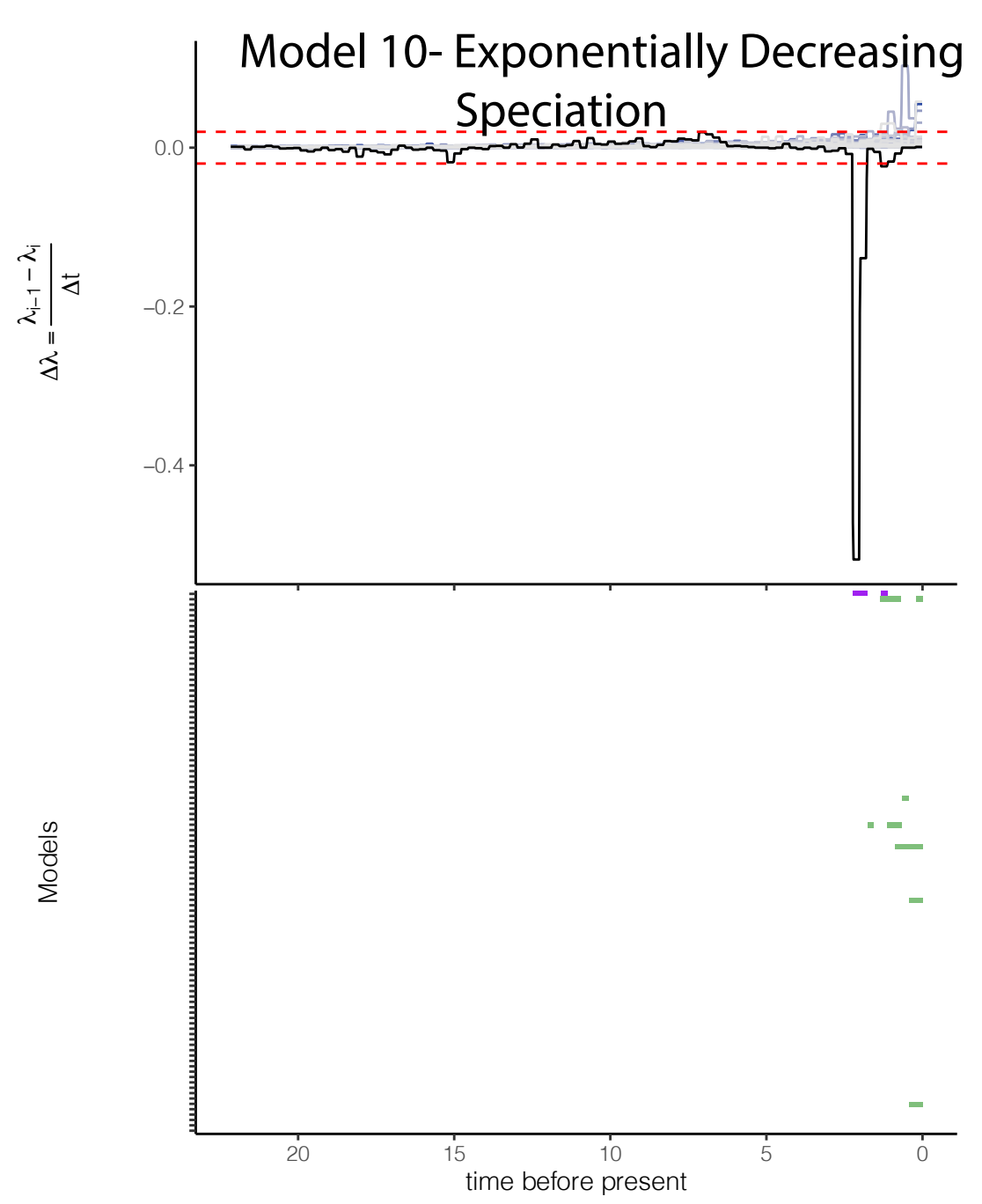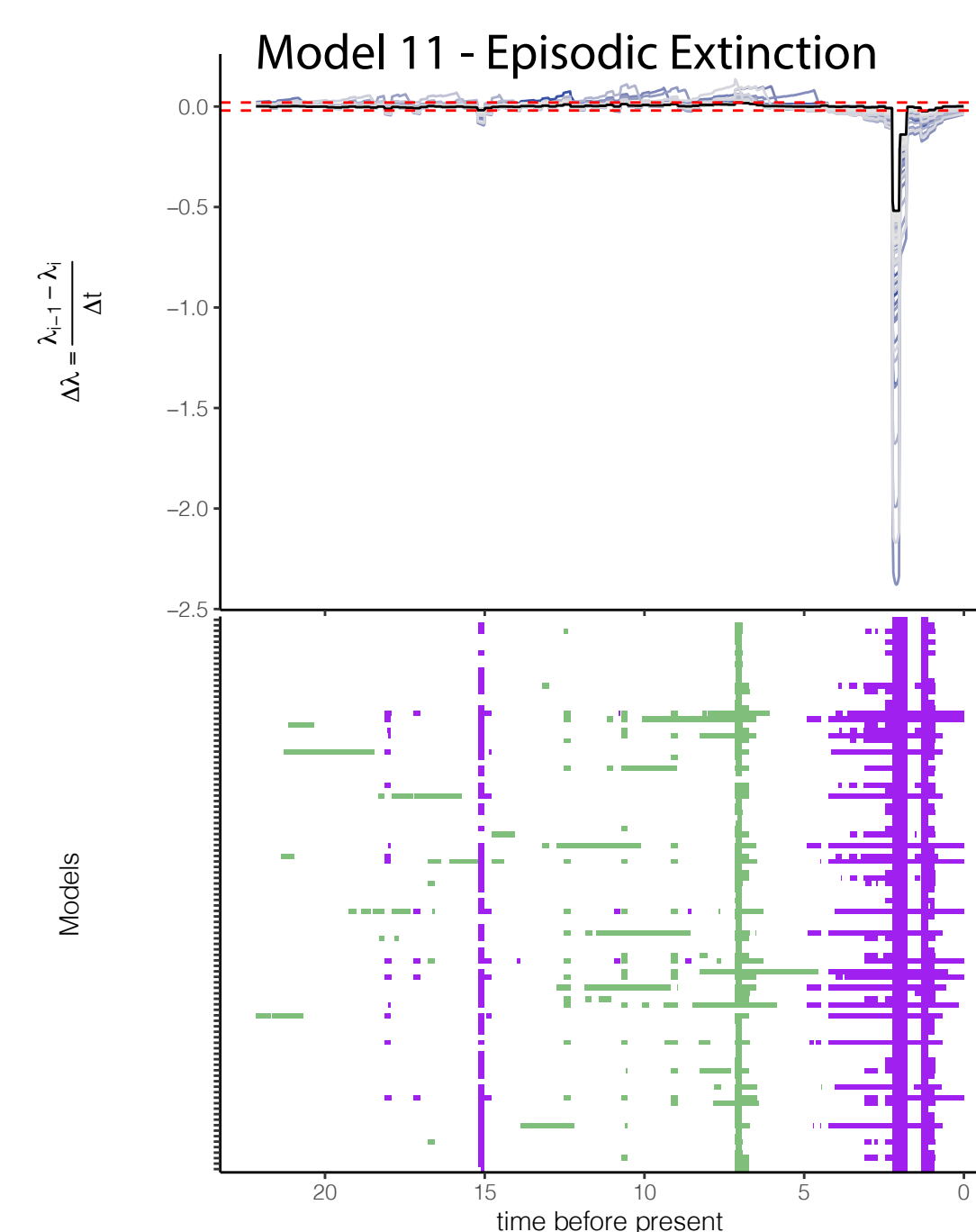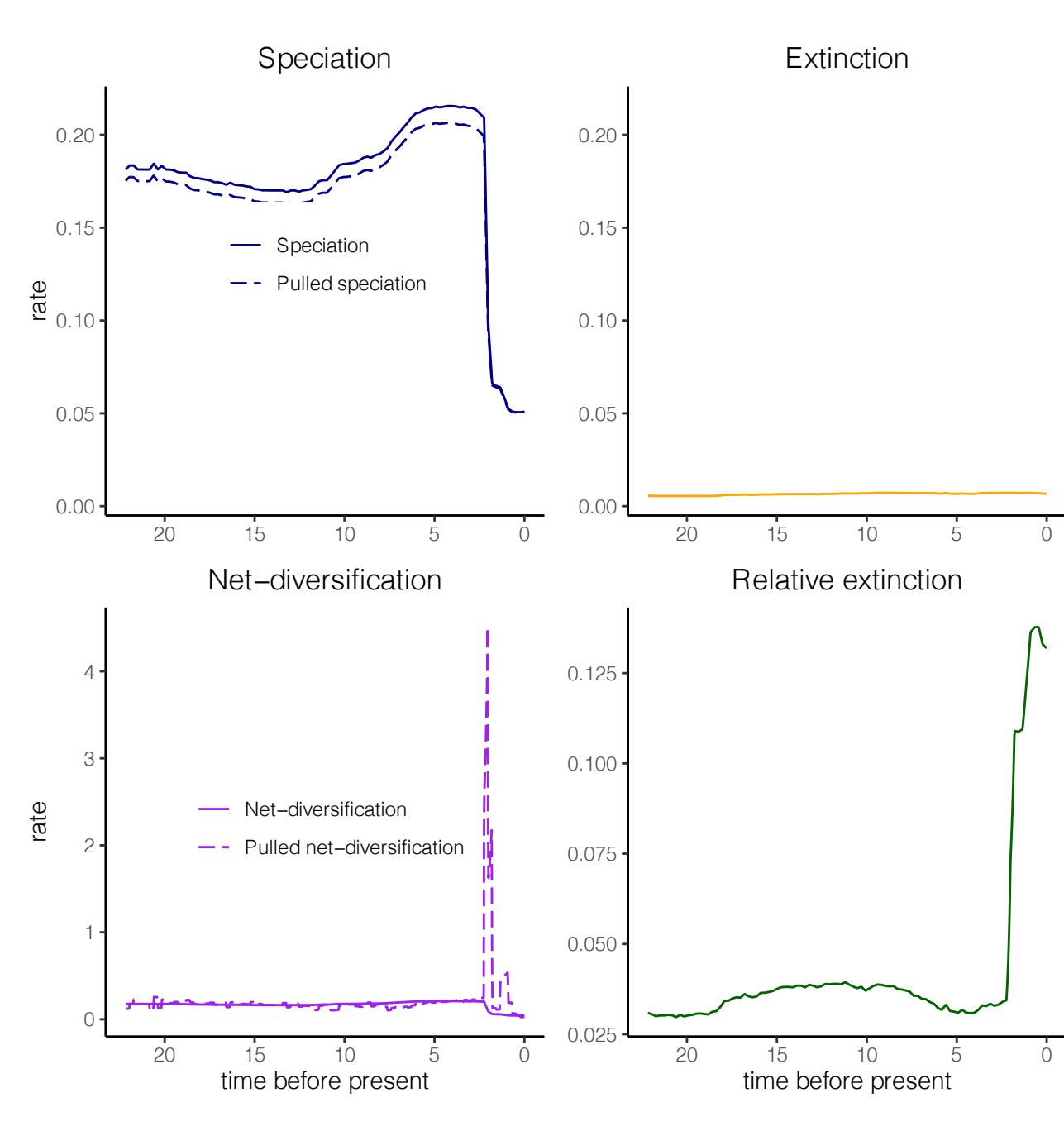

Supplement: Supplementary file 4 — Figure S2: [file ECE3-13-e10344-s003.pdf]

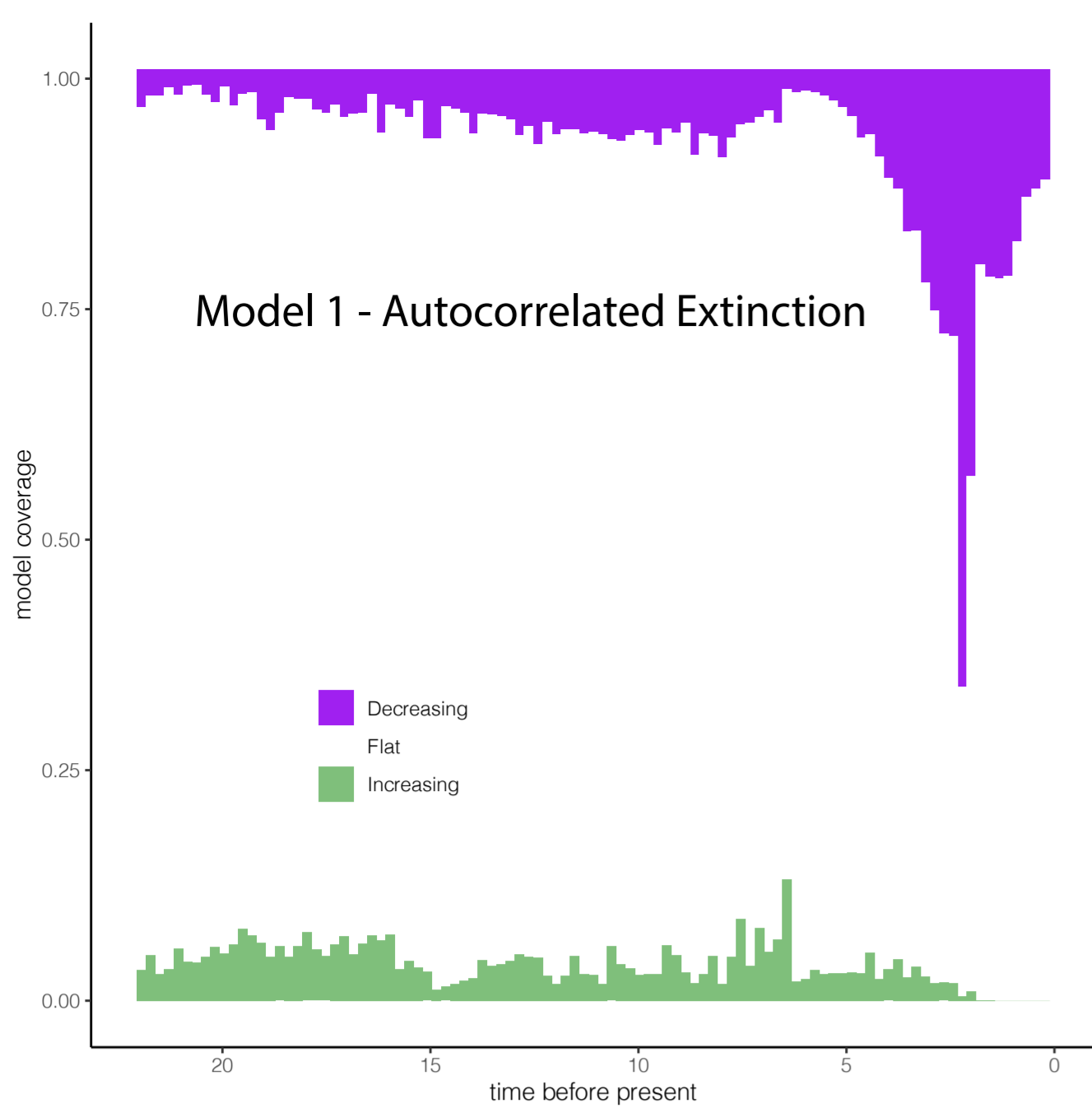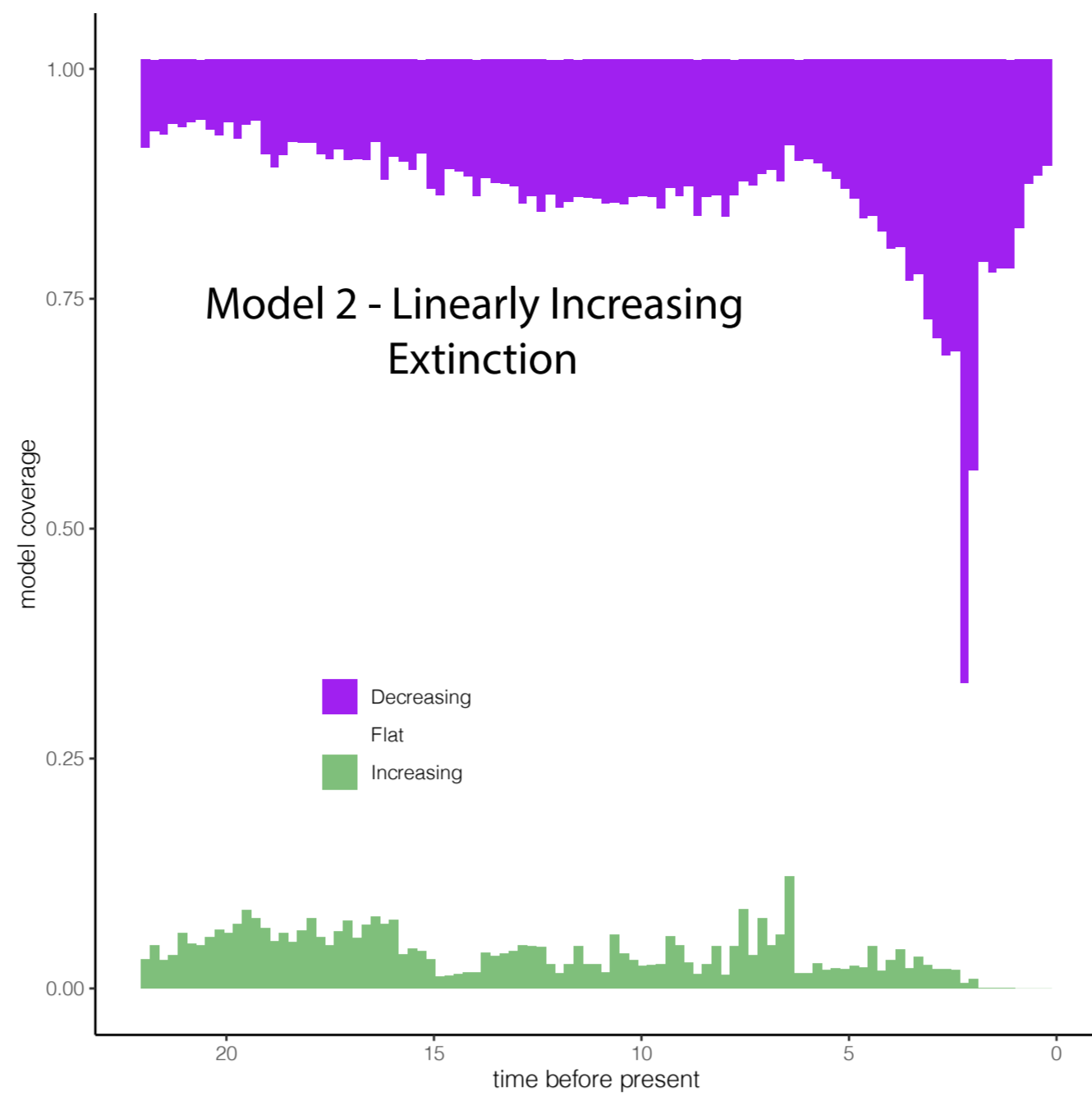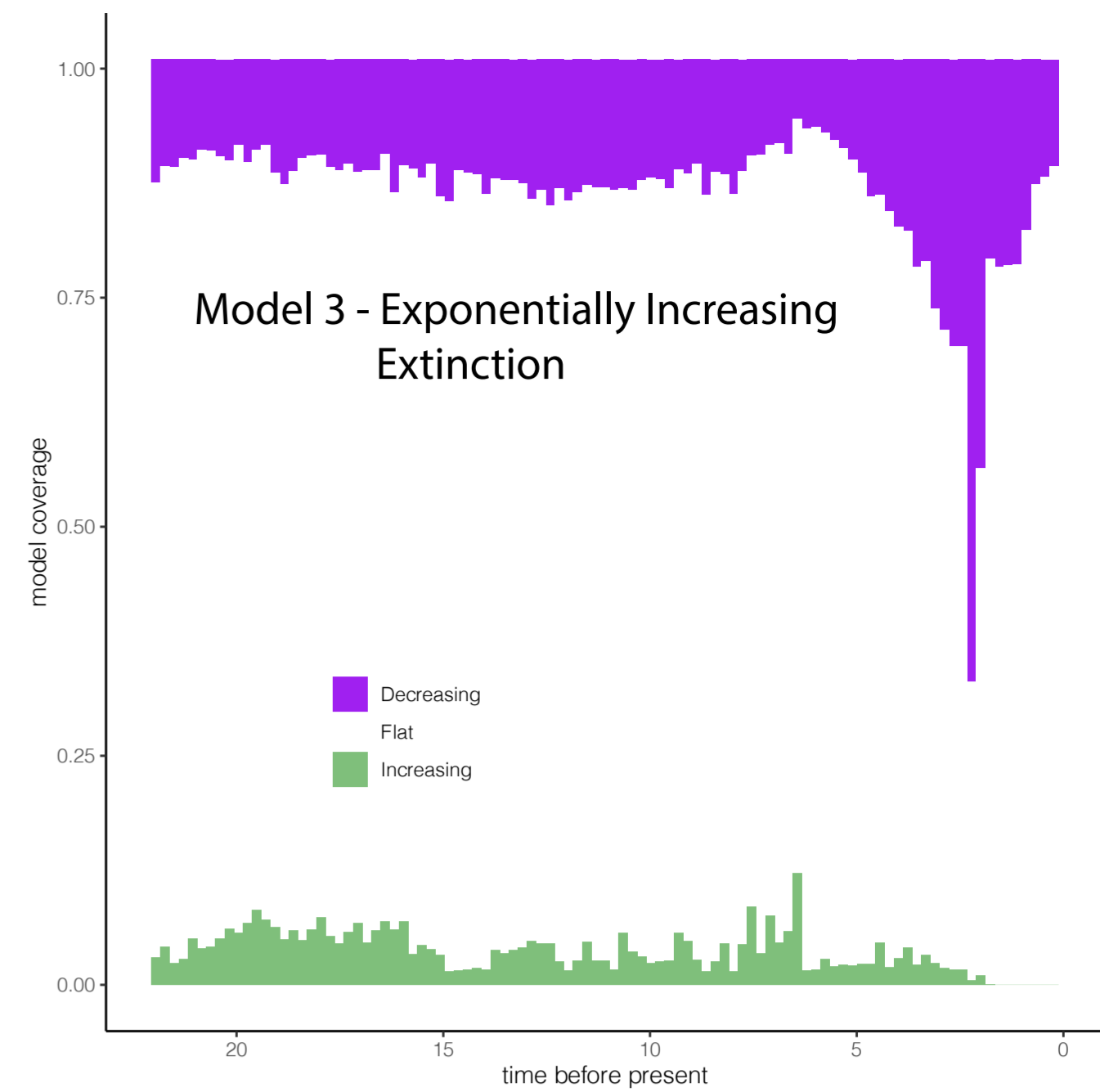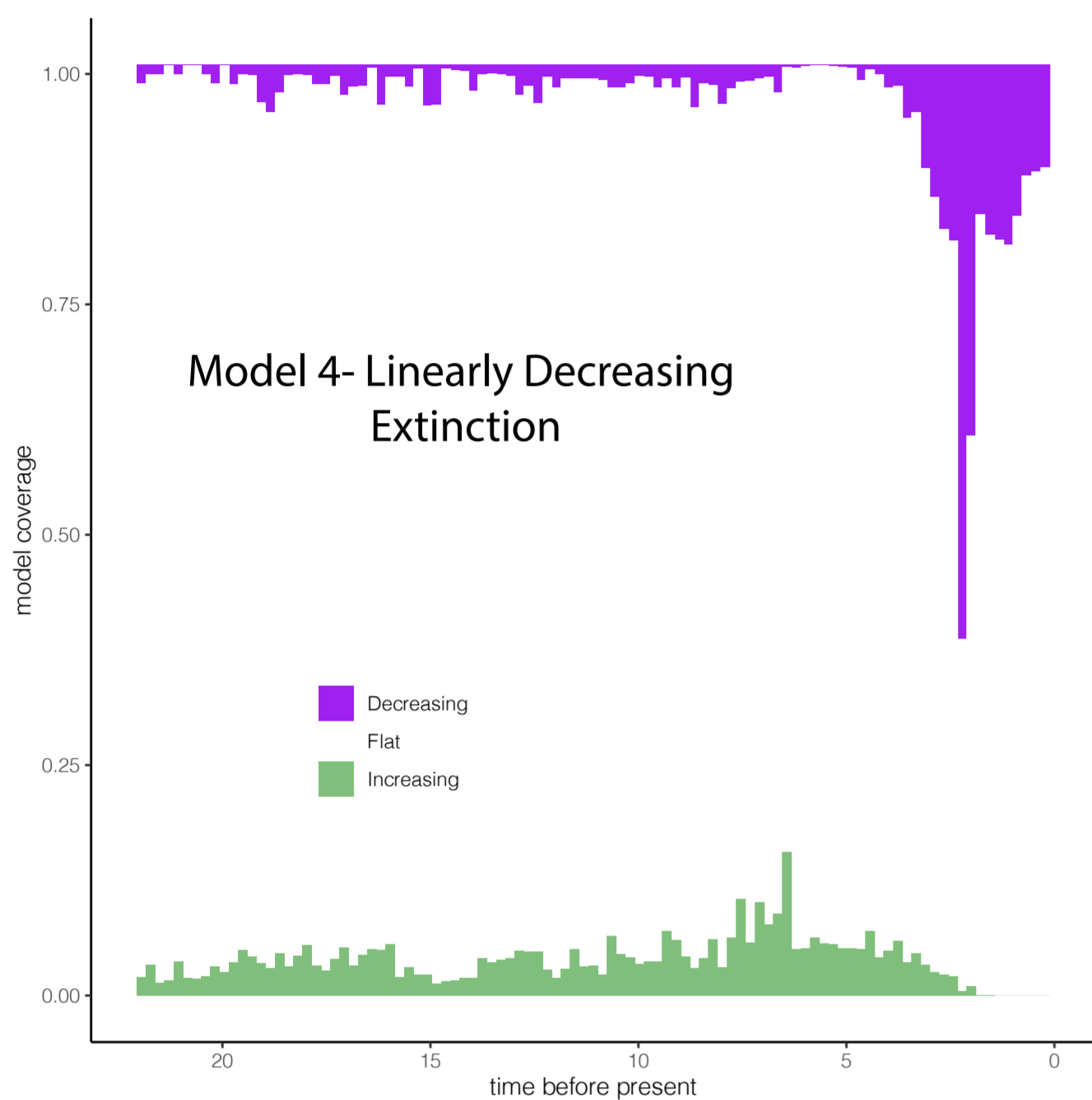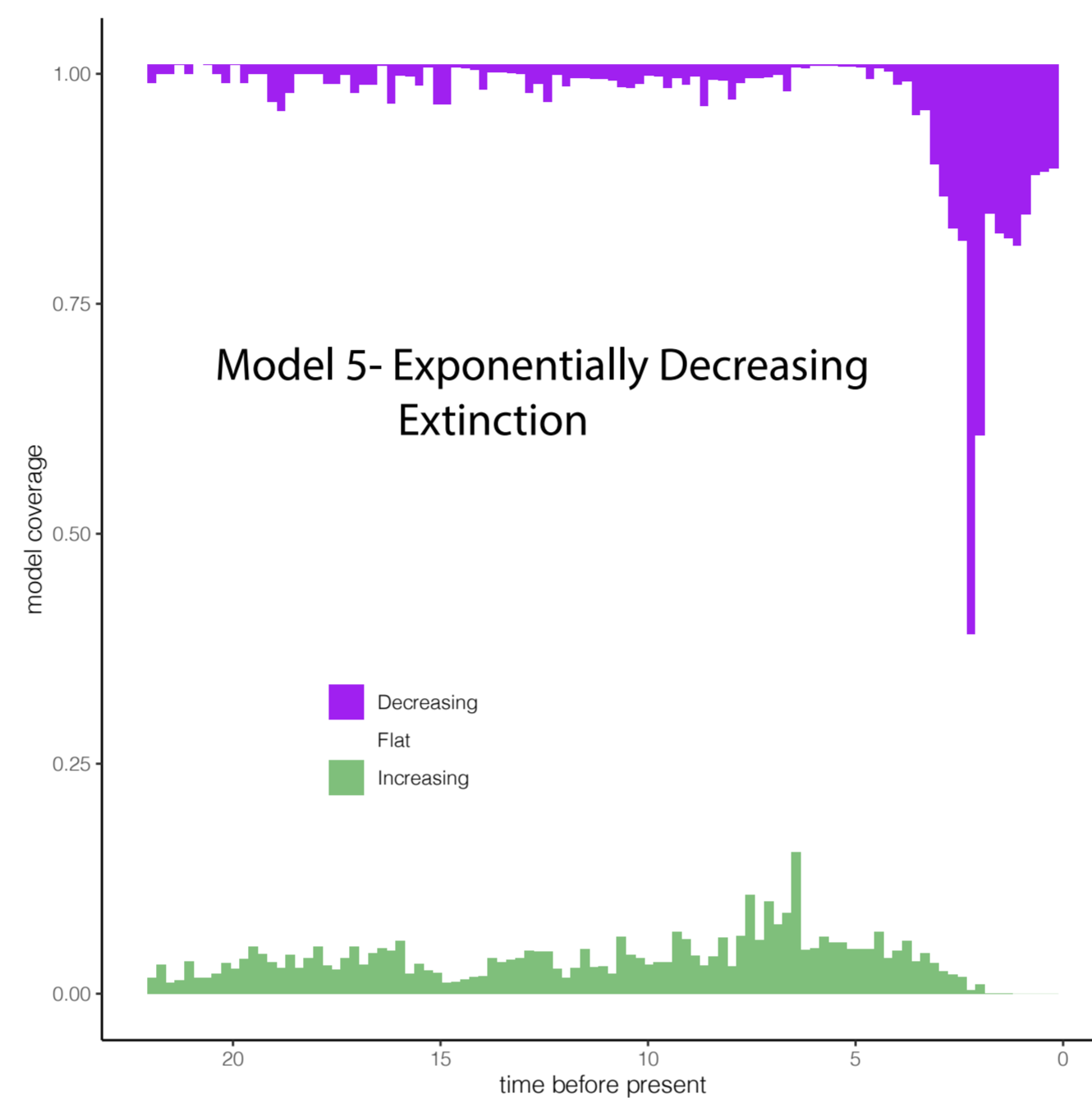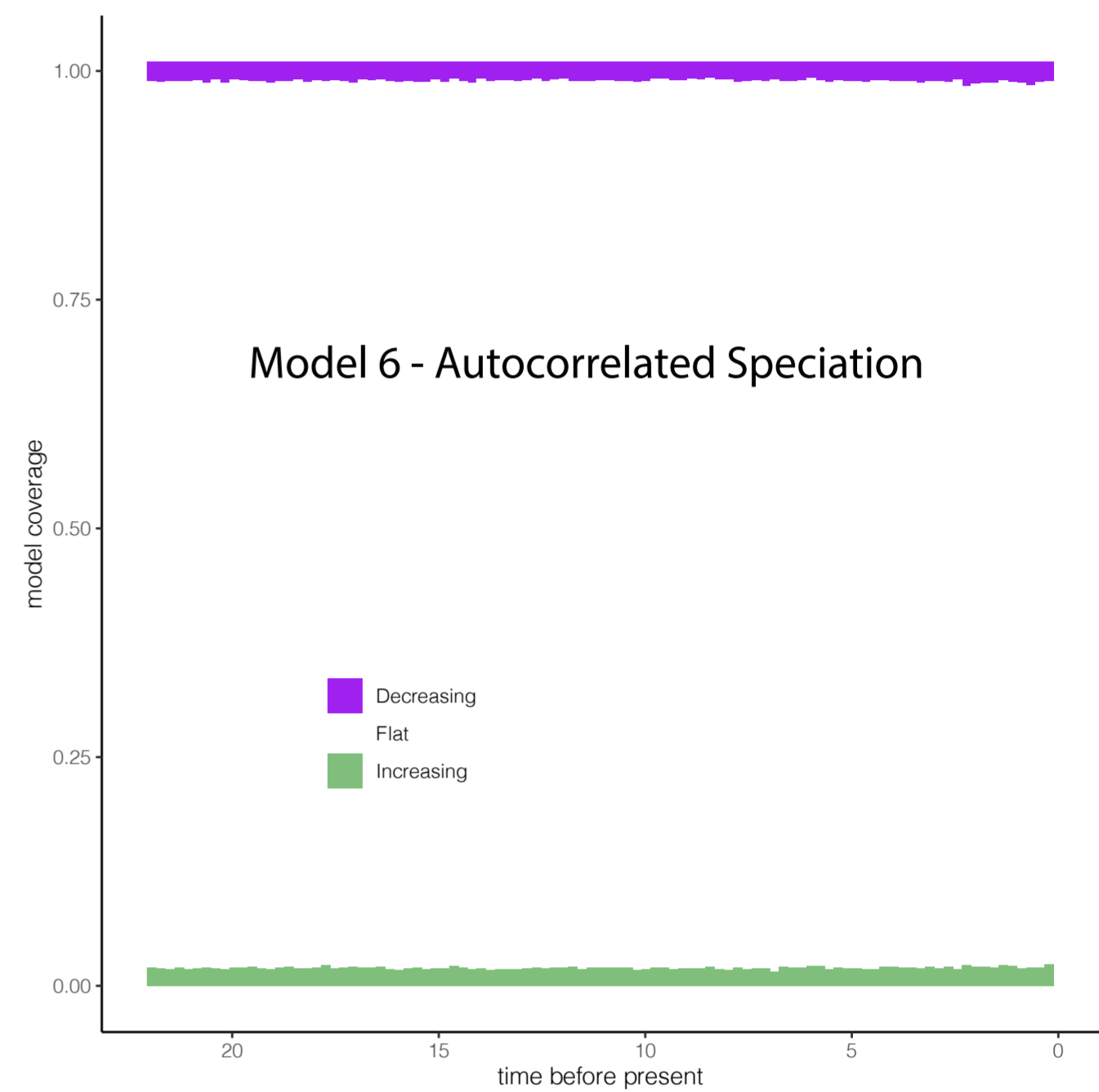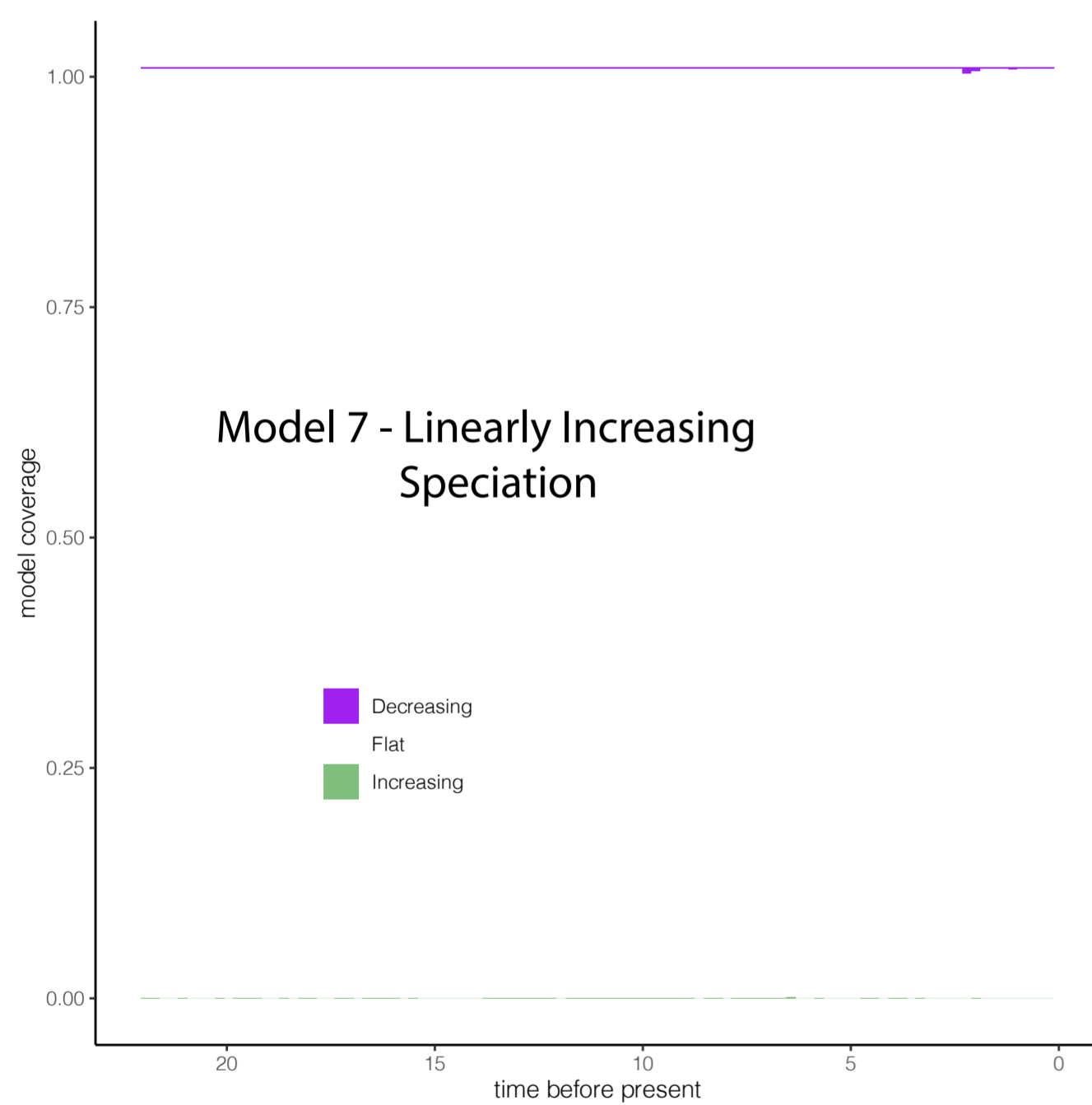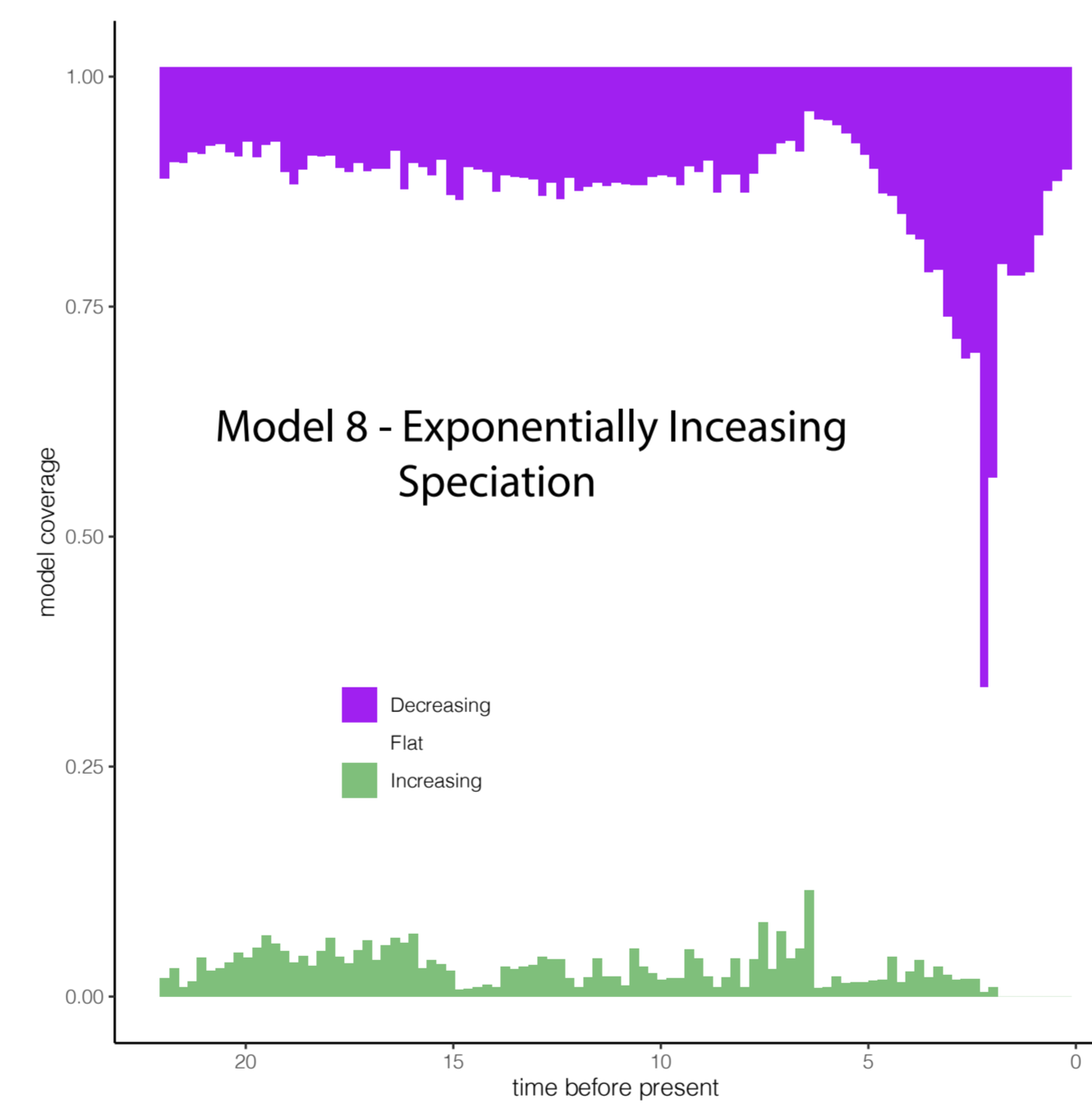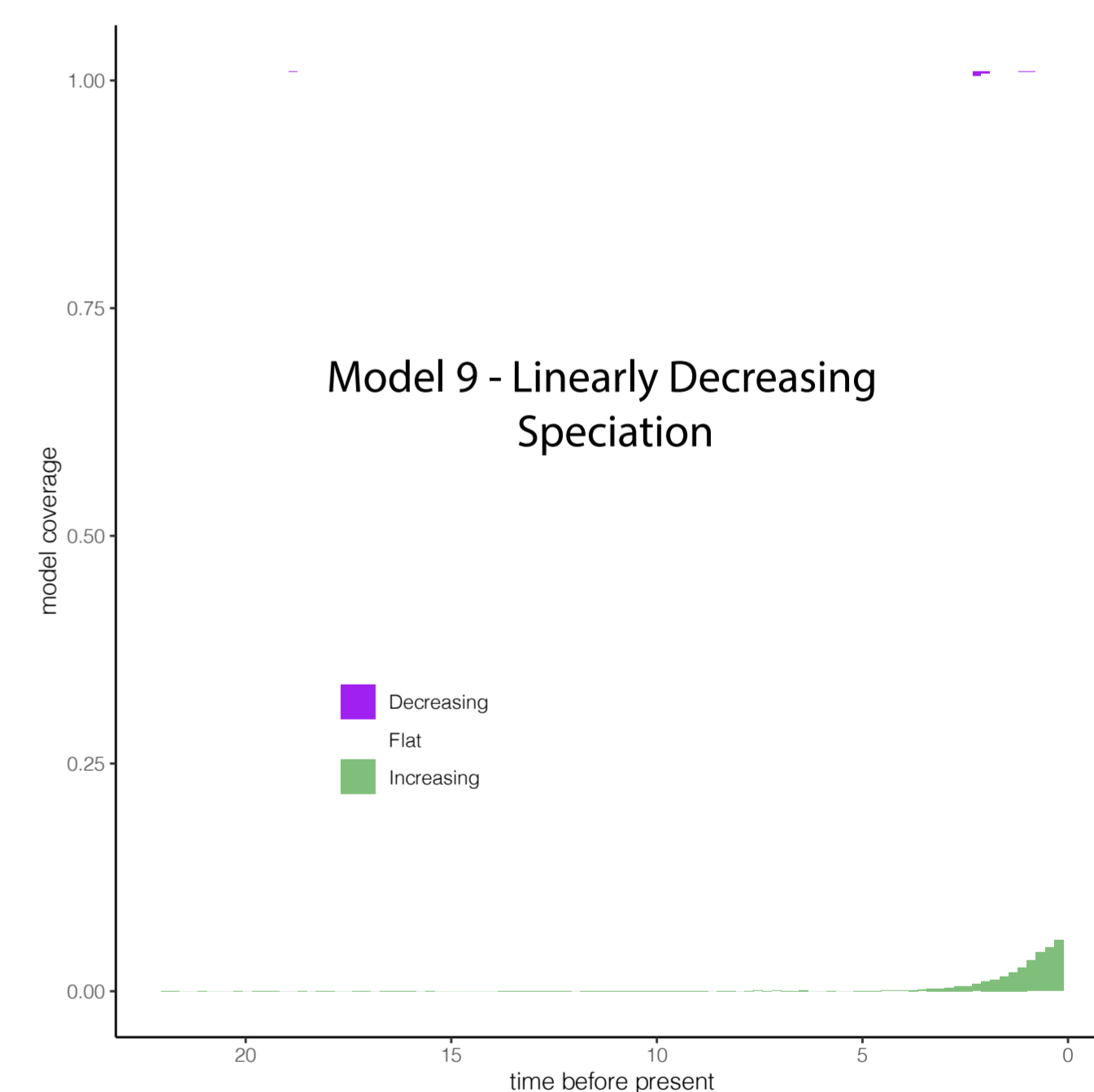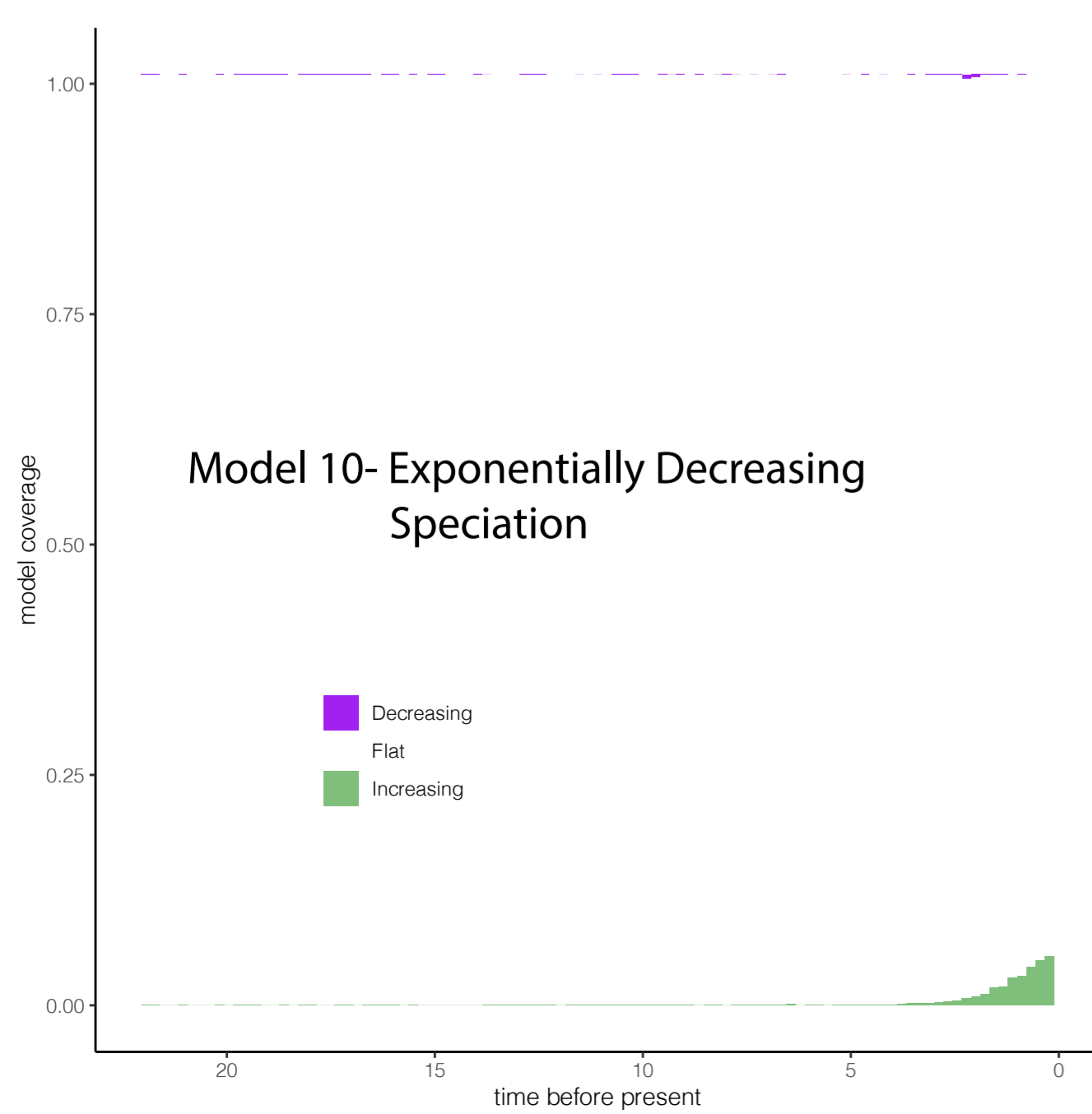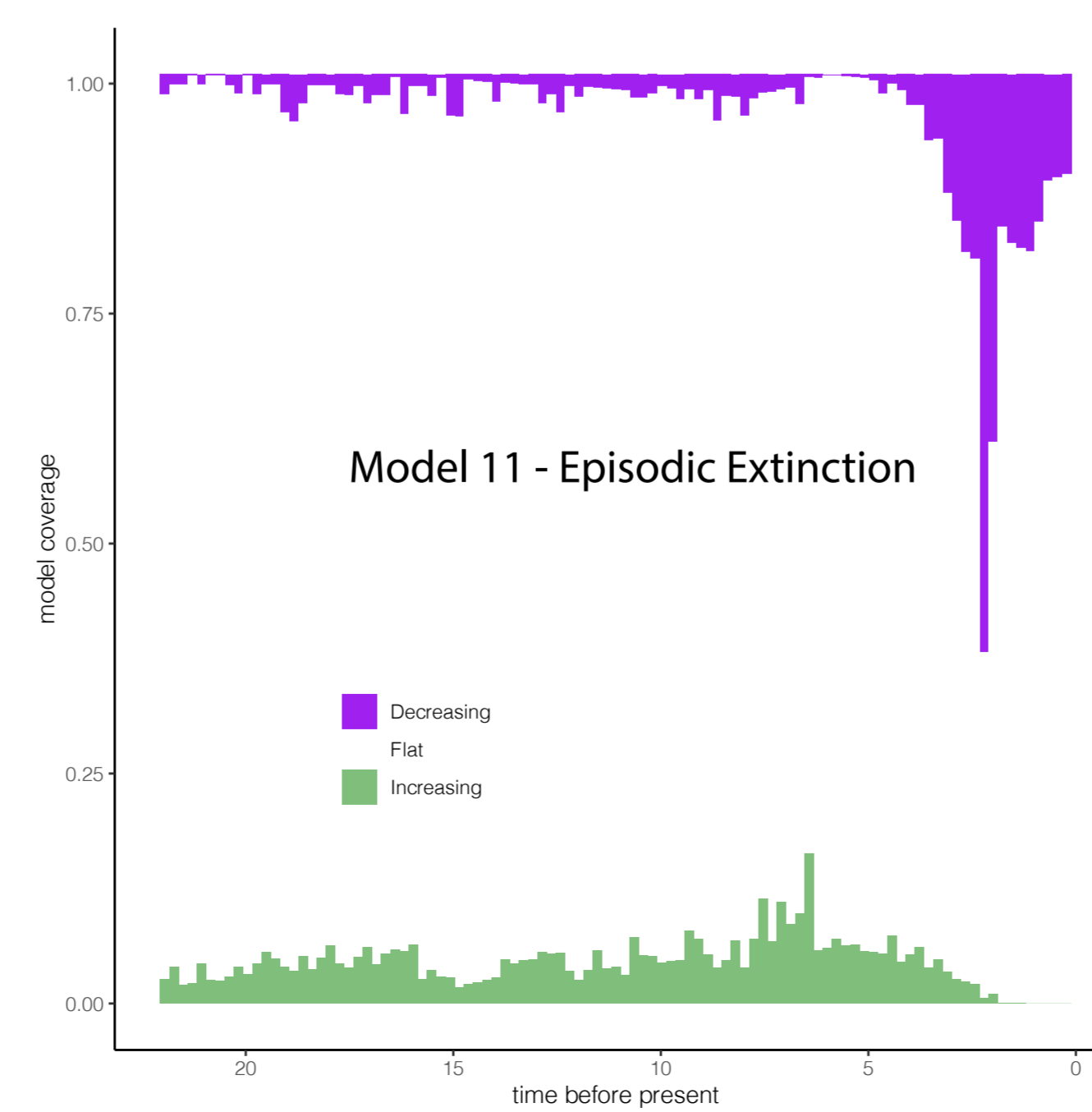

Supplement: Supplementary file 5 — Figure S3: [file ECE3-13-e10344-s001.pdf]
